# Formation of Hybrid Spherical Silica Particles Using a Novel Alkoxy-Functional Polysilsesquioxane Macromonomer as a Precursor in an Acid-Catalyzed Sol-Gel Process

**DOI:** 10.3390/ma18143357

**Published:** 2025-07-17

**Authors:** Anna Kowalewska, Kamila Majewska-Smolarek, Agata S. Herc, Sławomir Kaźmierski, Joanna Bojda

**Affiliations:** 1 Department of Polymeric Nano-Materials, Centre of Molecular and Macromolecular Studies, Polish Academy of Sciences, Sienkiewicza 112, 90-363 Łódź, Poland; kamila.majewska-smolarek@cbmm.lodz.pl (K.M.-S.); agatasherc@gmail.com (A.S.H.); joanna.bojda@cbmm.lodz.pl (J.B.); 2 Department of Structural Chemistry, Centre of Molecular and Macromolecular Studies, Polish Academy of Sciences, Sienkiewicza 112, 90-363 Łódź, Poland; slawomir.kazmierski@cbmm.lodz.pl

**Keywords:** hybrid silica, sol-gel, ladder polysilsesquioxane, microspheres, thermal stability, solid state NMR

## Abstract

The interest in macromolecular alkoxysilyl-functionalized hybrids (self-assembling or nanostructured), which could be used as precursors in biomimetic silica precipitation and for the synthesis of hollow spherical silica particles, is growing. Nevertheless, reports on all-organosilicon systems for bioinspired silica precipitation are scarce. Therefore, a new kind of polyalkoxysilane macromonomer–linear polysilsesquioxane (LPSQ) of ladder-like backbone, functionalized in side chains with trimethoxysilyl groups (LPSQ-R-Si(OMe)_3_), was designed following this approach. It was obtained by photoinitiated thiol-ene addition of 3-mercaptopropyltrimethoxysilane to the vinyl-functionalized polysilsesquioxane precursor, carried out in situ in tetraethoxysilane (TEOS). The mixture of LPSQ-R-Si(OMe)_3_ and TEOS (co-monomers) was used in a sol–gel process conducted under acidic conditions (0.5 M HCl/NaCl) in the presence of Pluronic^®^ F-127 triblock copolymer as a template. LPSQ-R-Si(OMe)_3_ played a key role for the formation of microparticles of a spherical shape that were formed under the applied conditions, while their size (as low as 3–4 µm) was controlled by the stirring rate. The hybrid materials were hydrophobic and showed good thermal and oxidative stability. Introduction of zinc acetate (Zn(OAc)_2_) as an additive in the sol–gel process influenced the pH of the reaction medium, which resulted in structural reinforcement of the hybrid microparticles owing to more effective condensation of silanol groups and a relative increase of the content of SiO_2_. The proposed method shows directions in designing the properties of hybrid materials and can be translated to other silicon–organic polymers and oligomers that could be used to produce hollow silica particles. The established role of various factors (macromonomer structure, pH, and stirring rate) allows for the modulation of particle morphology.

## 1. Introduction

Functional silica and doped silica materials obtained by sol-gel polymerization have found applications in adsorption and separation technologies, as protective coatings, materials for catalysis, environmental remediation, and controlled release (including biomedicine and biotechnology), as components of nanocomposites and ceramic membranes [1,2]. Synthesis of these materials under controlled conditions involves hydrolytic polycondensation of liquid precursors (silicon alkoxides) through simultaneous or sequential reversible reactions of hydrolysis and polycondensation (gelation) [3]. The relative rates of hydrolysis and condensation are sensitive to the parameters of the sol–gel process (chemical structure of the monomers, H_2_O content, pH of the medium, reaction temperature, use of cosolvents and surfactants, aging, and drying conditions) [4]. This makes it possible to adjust the structure of the resulting products, providing different functional hybrid materials with desired textural, chemical, and morphological properties (e.g., different particle size, pore geometry, and distribution) [5,6]. SiO_2_ particles with different structures can be obtained by polycondensation of Si(OH)_4_ in acidic, alkaline, or neutral environments (enzymatic biosilification) [7,8,9]. The formation of denser colloidal silica particles and colloidal gels is favored by base catalyzed condensation. Monodispersed silica microspheres are typically obtained in the NH_3_·H_2_O-catalyzed hydrolysis of TEOS in the presence of ethanol (the Stöber process [10]).

Controlled synthesis of functional silica aimed at obtaining materials for specific applications is gaining increasing interest [11]. Of particular importance are hollow silica microspheres, which can serve, for example, as drug delivery vessels [12,13,14]. A comparison of different methods of synthesizing hollow silica particles shows their potential advantages and disadvantages [15]. Hollow silica nanospheres and microspheres can be obtained using sacrificial hard [16] and soft [17] templates. The latter method is most suitable for the formation of mesoporous materials [including particles of spherical shape [18,19], but also aerogels [20], which were obtained with Pluronic^®^ F-127–a triblock copolymer poly(ethylene oxide)_99_–poly(propylene oxide)_69_–poly(ethylene oxide)_99_) as a surfactant].

Mesostructured silicas are often prepared with tetraethoxysilane (TEOS), while their organically modified analogues (organically modified silica, ORMOSIL) and more elaborated systems (including macromolecular alkoxysilyl-functionalized hybrids) are obtained with RSi(OR’)_3_ (R = an organic group; R’ = Me, Et) [21,22,23,24,25,26,27,28,29]. The geometry and morphological features of the precursors used in the synthesis of structured silica and ORMOSILs are of key importance. Structural differences affect the rate of hydrolysis of Si-OR’ groups. For example, in acid media, R_n_Si(OR’)_4−n_ hydrolyzes faster than Si(OR’)_4_. This fact is important when the sol–gel is conducted with a mixture of TEOS and R_n_Si(OR’)_4-n_. Depending on the pH of the reaction medium, self-condensates may be obtained along with the co-condensates. In porous ORMOSILs, different polarities of the organic part and the SiOH and SiOR’ groups can affect the chemical structure of the product. The hydrocarbon groups are located on the surface of pores, and the reactive groups are directed toward the inorganic wall.

Silica is often used as a carrier for metal oxide nanoparticles, including zinc oxide [30]. Incorporation in mesoporous silica can improve the characteristic optical and antimicrobial properties of ZnO [31]. It has also been shown that the encapsulation of ZnO nanoparticles in silica reduces their toxicity [32,33], increases their electrochemical detection capacity of Cd^2+^ ions [34] and photocatalytic activity [35], and improves their luminescence and stability [36]. Zinc acetate was shown to be an excellent precursor for ZnO nanoparticles. The reaction is usually conducted in the presence of a strong base [37,38] or under thermal decomposition conditions [39,40]. Monodisperse ZnO nanocrystals were also obtained via acid-catalyzed esterification of Zn(OAc)_2_ in the presence of *p*-toluene sulfonic acid [41]. However, with the use of hydrochloric acid, the situation is different. The double displacement reaction between Zn(OAc)_2_ and HCl is driven by the formation of the weak acid (CH_3_COOH) and yields zinc chloride (ZnCl_2_) that is highly soluble in water and dissociates into Zn^2+^ and Cl^−^ ions. The hydrolysis of ZnCl_2_ can lead to the formation of various mononuclear and polynuclear zinc hydroxide chloride species with the general formula of xZn(OH)_2_·yZnCl_2_·zH_2_O (e.g., zinc hydroxide chloride monohydrate (Zn(OH)Cl·H_2_O)), depending on the reaction temperature, pH, and the presence of ionic species (e.g., NaCl) [42,43,44]. Compounds such as Zn_5_(OH)_8_Cl_2_·H_2_O can be converted to crystalline ZnO by thermal decomposition [45].

In recent years, the attention of research has been directed towards biomimetic synthesis and nature-inspired materials and methods. In biomimetic chemistry of materials based on silica and organosilicon compounds, the main keyword is “silaffins”. Those peptides play a key role in diatom biomineralization and have inspired considerable interest in the design of self-assembling or nanostructured alkoxysilyl-functionalized hybrids as precursors in biomimetic silica precipitation and for the synthesis of hollow spherical silica particles [9,46,47,48,49,50]. Various macromolecular systems have been used in order to mimic the specific action of peptides in biosilica precipitation, including ionic polypeptides [51] and polyamines [52] but also combinations of amphiphilic block copolymers poly(methyl methacrylate-co-3-(trimethoxysilyl)propyl methacrylate)-b-poly(N-isopropylacrylamide) (P(MMA-co-MPMA)-b-PNIPAAm) and P(MMA-co-MPMA)-b-poly(2-(diethylamino)ethyl methacrylate) (P(MMA-co-MPMA)-b-PDEA) [23], 3-(trimethoxysilyl)propyl methacrylate grafted onto polypropylene [21] or fluorinated polymethylmethacrylate diblock copolymer [24], poly [3-(trimethoxysilyl)propyl methacrylates] of various architecture [25,28] or 1-vinyl-1,2,4-triazole(trimethoxysilyl)methylmethacrylate copolymers [27]. The common feature of the non-ionic macromolecular systems is the ability for self-organization and the presence of an organic polymer chain as the core, to which trialkoxysilyl groups are attached by various types of bonds and interactions.

**Scheme 1:**
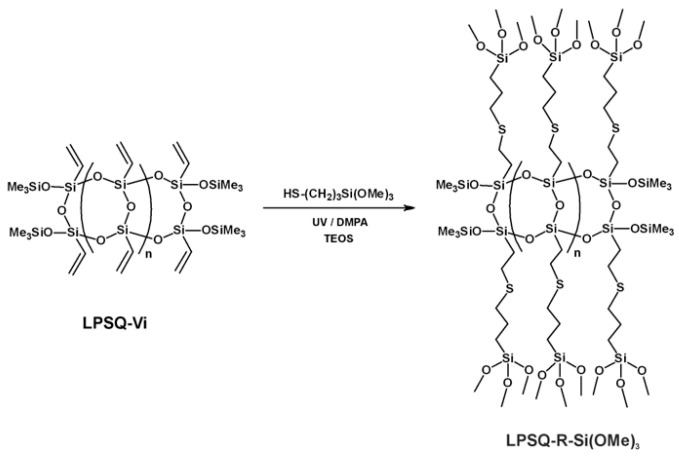
Synthesis of oligomeric LPSQ-R-Si(OMe)_3_ terminated with trimethoxysilyl groups.

However, there is a general lack of information on all-organosilicon systems for bioinspired silica precipitation. In this report, we present results obtained with unique polyalkoxysilane macromonomers–linear polysilsesquioxanes (LPSQ) functionalized with trimethoxysilyl groups in their side chains (LPSQ-R-Si(OMe)_3_) (Scheme 1). LPSQ macromolecules with a ladder-like structure of the main backbone offer better solubility, processability, mechanical stability, and heat resistance, compared to polyhedral oligomeric silsesquioxanes (POSS). These properties provide them great potential for durable coatings and sealants, but also as innovative, advanced hybrid materials for electronics and energy storage [53,54]. Our earlier studies on the synthesis and modification of ladder-like poly(vinylsilsesquioxanes) (LPSQ-Vi) have shown that these macromolecules can be valuable precursors of hybrid superhydrophilic or highly emissive polymers that can be used in surface modification [55,56,57], materials engineering [58,59,60], and optoelectronics [61,62].

The properties of polymers derived from LPSQ-Vi reflect their unique structural features. The well-defined arrangement of siloxane bonds increases the rigidity of LPSQ (compared to single-chain polysiloxanes of similar chain length) and ensures a regular distribution of functional substituents along the polymer chain. This prompted us to convert LPSQ-Vi into a macromonomer multifunctionalized with alkoxysilyl groups–similar to those designed for biomimetic silica precipitation–and study its behaviour in the sol-gel reaction. We chose to test acidic conditions as a method that yields fewer branched oligomers. This would be beneficial for the initial stages of microparticle formation and less detrimental to the siloxane bonds in LPSQ.

LPSQ-R-Si(OMe)_3_ was obtained by a click-type reaction–thiol-ene addition of 3-mercaptopropyltrimethoxysilane to LPSQ-Vi. The photoinitiated reaction was conducted in tetraethoxysilane (TEOS), which was used as both solvent and co-monomer in the subsequent synthesis step (sol–gel process). The shape of microparticles obtained with LPSQ-R-Si(OMe)_3_ in the sol–gel reaction conducted in 0.5 M HCl, in the presence of NaCl and Pluronic^®^ F-127, was spherical. The pH of the sol–gel reaction was modulated by the addition of Zn(OAc)_2_ and in situ formation of xZn(OH)_2_·yZnCl_2_·zH_2_O. Comparative analysis of the results obtained with LPSQ-R-Si(OMe)_3_ showed that the presence of these unique macromolecules as co-macromonomers, along with Si(OEt)_4_, played a key role in the sol–gel process. Previously, it was shown that spherical particles can be obtained with the TEOS/Pluronic^®^ F-127/HCl system, but only under high acid concentrations, while lowering the amount of HCl yielded monolithic silica [19]. We proved that under the conditions used, the formation of microspheres should be associated with the presence of LPSQ-R-Si(OMe)_3_, while their size was mainly controlled by the stirring rate. The use of zinc acetate as an additive in the sol–gel reaction increased the pH of the reaction medium and caused an increase in silanol condensation, resulting in structural strengthening of the hybrid silica and an increase in their size.

## 2. Materials and Methods

### 2.1. Materials

Commercial available reagents: tetraethoxysilane (TEOS, ABCR GmbH, Karlsruhe, Germany, 99%), 3-mercaptopropyltrimethoxysilane (ABCR GmbH, Karlsruhe, Germany, 95%), Pluronic^®^ F-127 (Sigma-Aldrich, St. Louis, MO, USA), zinc acetate dihydrate (Zn(OAc)_2_, Sigma-Aldrich, St. Louis, MO, USA, >98%), hydrochloric acid (Avantor Performance Materials, Gliwice, Poland, 0.5 mol/L volumetric solution), sodium chloride (Chempur, Piekary Śląskie, Poland, pure p.a.), 2,2-dimethoxy-2-phenylacetophenone (DMPA, Acros Organics, Geel, Belgium, 99%) were used as received. The linear poly(vinylsilsesquioxane) precursor (LPSQ-Vi, M_n_ = 1 kg/mol, M_w_/M_n_ = 1.4 (SEC/RI) was obtained as earlier described [63].

### 2.2. Synthetic Methodology

#### 2.2.1. Synthesis of LPSQ-R-Si(OMe)_3_

Oligomeric polysilsesquioxanes LPSQ-R-Si(OMe)_3_ of double-strand backbone terminated with trimethoxylsilyl groups were prepared by photoinitiated thiol-ene addition of 3-mercaptopropyltrimethoxysilane to LPSQ-Vi in the presence of DMPA as a radical initiator (Scheme 1), analogously to earlier published procedures [55,64]. TEOS was used as a solvent in this reaction.

In an exemplary procedure, the thiol derivative (0.81 mL, 0.856 g, 4.36 mmol), LPSQ–Vi (0.4 g, 4.36 mmol Vi), and TEOS (1.0 mL, 0.933 g, 4.48 mmol) were placed in a quartz vessel. DMPA (0.026 g, 0.1 mmol) was added with stirring to the solution of reagents. The mixture was irradiated for 15 min with UV light (λ = 356 nm). The mixture of products was used in the next step without any work-up. Smaller amounts of TEOS were used in experiments with a lower ratio of [TEOS]_0_/[LPSQ-R-Si(OMe)_3_]_0_.

#### 2.2.2. Sol–Gel Reaction

The sol-gel process was conducted by using non-ionic surfactant as the structure-directing agent. The basic procedure was adapted from the literature [65]. In an exemplary preparation, 0.92 g of Pluronic^®^ F-127 and 2.76 g of NaCl were dissolved in 36.8 mL of 0.5 mol/L HCl solution with stirring at 40 °C. The pH of acidic solutions containing NaCl, F-127, and Zn(OAc)_2_ was measured with a pH meter CP-501 (Elmetron, Zabrze, Poland) with an integrated pH probe (EPP-1 type). An increase in pH of the acidic solutions was observed on the addition of Zn(OAc)_2_ (for example, pH of the reaction medium in experiments I-2, II-2, and II-1 was equal to 0.45, 0.89, and 3.30, respectively).

At the same time, the mixture of alkoxysilane reagents was prepared (amounts provided in Table 1, Section 2.2.1). TEOS or the mixture of co-monomers (LPSQ-R-Si(OMe)_3_ and TEOS) was added dropwise with stirring to the homogenous solution containing F-127 at 40 °C. The reaction mixture was heated at 40 °C over the next 20 h. Six different stirring procedures (Table 1) were applied:

A: Magnetic stirring (70 rpm) over the whole reaction time of 20 h;
B: Magnetic stirring (70 rpm) only during dropping in the alkoxysilanes;
C: Magnetic stirring (600 rpm) during dropping in the alkoxysilanes and for additional 30 min afterwards;
D: Fast stirring (20,000 rpm) with a homogeniser (High Shear Homogeniser Unidrive X 1000 CAT, CAT Scientific, Paso Robles, CA, USA) during addition of alkoxysilanes and for additional for 3 min;
E: Magnetic stirring at highest rate (1500 rpm) during dropping in the alkoxysilanes and for additional 30 min afterwards;
F: Slow stirring (4000 rpm) with the homogeniser during addition of alkoxysilanes and for additional 10 min.

In experiments conducted with TEOS, the turbidity signifying formation of silica particles occurred only after about 1.5–2 h of heating at 40 °C. In contrast, in experiments with LPSQ-R-Si(OMe)_3_/TEOS, we observed that precipitation of white particles occurred after about 5 min after the addition of alkoxysilane co-monomers was completed. Despite this, the reaction mixtures were heated for 2 h more at 40 °C. The solid products were filtered on blotting paper strainers, washed with distilled water until neutralized, and air-dried at room temperature.

In experiments of type II and IV, Zn(OAc)_2_ was added to the homogeneous solution of Pluronic^®^ F-127/HCl/NaCl. After about 10 min at 40 °C, Zn(OAc)_2_ dissolved completely in this mixture and the alkoxysilanes were added dropwise (with stirring as above). The presence of Zn^2+^ ions and the change of pH generally did not affect the length of the induction period before the appearance of turbidity in reactions conducted with TEOS alone, or with the mixtures of alkoxysilane co-monomers as precursors.

#### 2.2.3. Pyrolysis of the Hybrid Silicas

Samples of silica/hybrid silica were placed in ceramic combustion boats and were placed in a high-temperature tube furnace (Nabertherm RHTH 120/300/18, Nabertherm GmbH, Lilienthal, Germany). Pyrolysis was conducted at 217 °C in an air atmosphere. Heating rate was 5 °C/min. The samples were kept for 2 h at the target temperature. Then, heating was switched off, and the sample was allowed to cool to room temperature in the flowing argon atmosphere. After cooling, the samples were weighed and analyzed. Sample III-1 was subjected twice to this thermal treatment.

### 2.3. Analytic Methods

#### 2.3.1. NMR Spectroscopic Methods

Liquid-state measurements were performed in C_6_D_6_ as the deuterated solvent, on Bruker Avance NEO 400 NMR spectrometer (Rheinstetten, Germany), operating at 400.15 MHz for ^1^H and 100.63 for ^13^C and 79,50 for ^29^Si, and operating with SampleCase Plus autosampler. The spectrometer is equipped with 5 mm high-resolution dual-channel ^2^H/^1^H(^19^F)/BB i-Probe^®^ with and z-gradient coil capable of tuning to nuclei ^19^F/^31^P-^199^Hg and ^17^O-^109^Ag on the BB channel with automatic tuning and matching system (ATMA). Spectrometer is operated using TopSpin 4.1.1 program running under Windows 10 Pro operating system, and spectra were recorded under IconNMR automation program, version 5.2.1 (Build 11).

For ^1^H spectra, 32 scans were accumulated per FID of 64 K data points with 1s relaxation delay (D1), and spectral width was set to 8012 Hz (16 ppm), with results in 3.99 s of acquisition time (AQ). Original pulse program zg30 from Bruker pulse program library was used. FIDs were zero-filled twice and apodized with LB function (0.3 Hz) prior to Fourier transformation.

To obtain quantitative results, both ^13^C and ^29^Si spectra were recorded with inverse-gated decoupling technique with 30-degree observe pulse (zgig30 pulse program). Between 2048 and 5120 scans were accumulated for each spectrum with 64K data points of FIDs with spectral width of 250 ppm. WALTZ-16 sequence during acquisition time for proton decoupling and delay of 60 s for relaxation were applied. Chemical shift for ^13^C was calibrated using signal of solvent (^13^C δ_C6D6_ = 128.7 ppm) ppm, and ^29^Si spectra were referenced externally, with respect to TMS (^29^Si δ_TMS_ = 0.0 ppm).

The solid-state ^13^C and ^29^Si MAS experiments were conducted on Bruker Avance III 400WB spectrometer (Rheinstetten, Germany), operating at 400.19 for ^1^H and 100.63 MHz and 79.506 MHz for ^13^C and ^29^Si, respectively. Spectra were acquired with MAS probe head using 4-mm ZrO_2_ rotors.

Both ^13^C and ^29^Si spectra were registered using High-Power Decoupling (HP Dec) method with relaxation delay of 100 s and swftppm12 proton decoupling sequence applied during acquisition time. MAS frequency was set at 8000 Hz, and 512 scans per spectrum with a time domain size of 4000 data points were accumulated.

For ^13^C MAS spectra, a sample of native glycine was used for pulse calibration and as an external chemical shift reference (δ_C=O_ = 176.5 ppm). For ^29^Si MAS spectra, a sample of Q_8_M_8_ was used for pulse calibration and as an external chemical shift reference (δ = 11.7 ppm for OSi(CH_3_)_3_ group).

All spectra were acquired and processed using TopSpin 2.1 program.

#### 2.3.2. Thermogravimetric Analysis (TGA) and Differential Thermal Analysis (DTA)

Thermogravimetric analysis (TGA) and Differential Thermal Analysis (DTA) were conducted using a thermogravimetric analyser (TGA Q50 V20.13 Build 39. Universal V4.5A TA Instruments, New Castle, DE, USA) at 10 °C/min from 20 to 600 °C under air or nitrogen atmosphere.

TGA and DTA were also used in an experiment to evaluate the removal efficiency of surfactant F-123 during pyrolysis in an oven at 217 °C (for 2 h in an air atmosphere). Isothermal decomposition of a selected sample (III-1) was conducted using the thermogravimetric analyser at 217 °C under air atmosphere. The heating (10 °C/min ramp) was started at room temperature until the isotherm was reached. The sample was kept at the chosen temperature until no weight decrease was observed.

#### 2.3.3. Scanning Electron Microscopy (SEM and SEM-EDS)

The surface morphology of the studied samples was characterized using scanning electron microscopy (SEM) (Jeol 5500LV, Tokyo, Japan) operating in the high vacuum mode and at an accelerating voltage of 10 kV. Samples were mounted on brass stubs using double-sided adhesive tape and sputtered with gold using a Jeol Fine Coater 1200, Tokyo, Japan.

Scanning electron microscopy equipped with energy dispersive spectroscopy (SEM-EDS, Jeol JSM-6010LA, Tokyo, Japan) was performed in the high vacuum mode at an accelerating voltage of 15 kV. Prior to analysis, the surfaces of specimens were sputter-coated with carbon using a Q150R ES coater (Quorum Technologies, Lewes, UK).

#### 2.3.4. Wide-Angle X-Ray Diffraction

Wide-angle X-ray scattering (WAXS) measurements of selected samples (II-1 and IV-4, Table 1) were performed on a computer-controlled goniometer coupled with a CuKα radiation source (λ = 0.154 nm), operating at 30 kV and 50 mA (Panalytical B.V., Almelo, The Netherlands). The 2Θ scans were collected in reflection geometry mode in a 2θ range from 10 to 40 with a step of 0.05° at room temperature.

## 3. Results

### 3.1. Synthesis of LPSQ-R-Si(OMe)_3_

LPSQ-Vi was transformed into LPSQ-R-Si(OMe)_3_ by photoinitiated addition of 3-mercaptopropyltrimethoxysilane, with 2,2-dimethoxy-2-phenylacetophenone (DMPA, [DMPA]_0_/[Vi]_0_ = 0.023) under UV light (*λ* = 356 nm). The addition of 3-mercaptopropyltrimethoxysilane to unsaturated C-C bonds has been previously used to functionalize silanes [66], POSS [67,68,69,70], as well as other organosilicon compounds and materials [71,72,73]. The innovation in the method we proposed in this report is the use of tetraethoxysilane (TEOS) as a solvent for thiol-ene addition. This procedure can be widely employed to prepare a range of alkoxy functional macromonomers for use in sol–gel (acid- and base-catalyzed) reactions.

**Figure 1:**
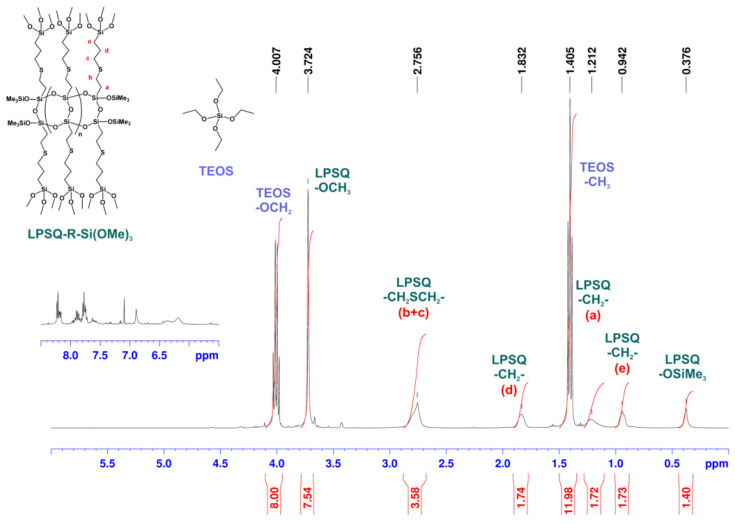
^1^H NMR (C_6_D_6_) of a reaction mixture sample taken directly from the crucible (inset: enlarged region of the spectrum with traces of vinyl groups and the products of degradation of DMPA). Symbols a–e indicate different types of methylene protons in LPSQ-R-Si(OMe)_3_.

**Figure 2:**
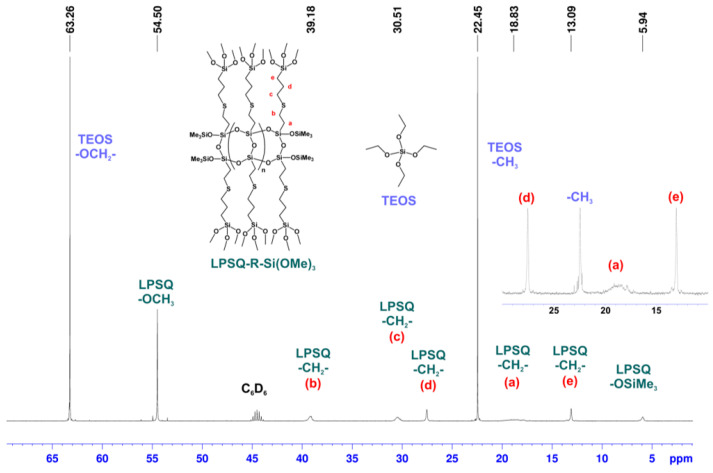
^13^C NMR(C_6_D_6_) of a reaction mixture sample taken directly from the crucible (inset: the region within 10–30 ppm enlarged to show better the broad peak corresponding to -CH_2_- (a). Symbols b–e indicate other types of methylene protons in LPSQ-R-Si(OMe)_3_.

**Figure 3:**
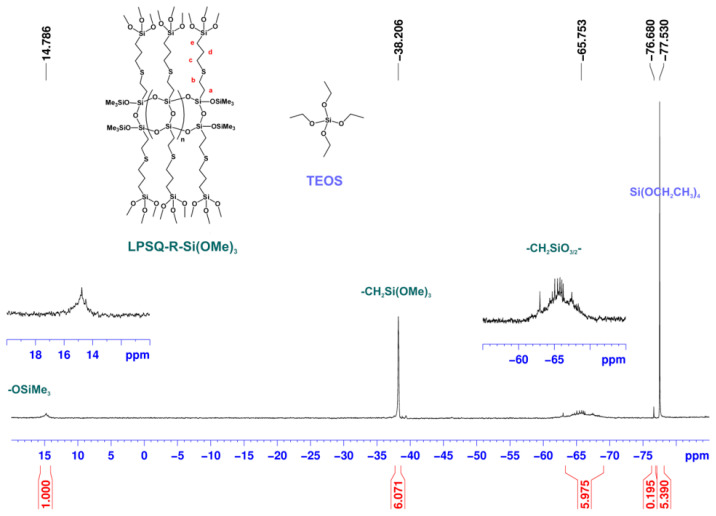
^29^Si NMR(C_6_D_6_) of a reaction mixture sample taken directly from the crucible (insets: Si atoms in the main backbone (T-type at about −65 ppm) and end groups (M-type at about 15 ppm). Symbols a–e indicate different types of methylene protons in LPSQ-R-Si(OMe)_3_.

NMR spectroscopy (Figure 1, Figure 2 and Figure 3) proved that this approach yielded the desired mixture of LPSQ-R-Si(OMe)_3_ and TEOS, which can be further used as a co-reagent composition in the sol–gel process under acidic conditions and in the presence of Pluronic^®^ F-127. The molar ratio [Vi]_0_/[HS]_0_ = 1.01 was used to ensure that no free HS-(CH_2_)_3_Si(OMe)_3_ remained in the reaction mixture. The molar ratio [TEOS]_0_/[LPSQ-R-Si(OMe)_3_]_0_ was adjusted to match that designed for the sol–gel experiments with a different concentration of TEOS. This approach eliminated the need for the purification step and protected the methoxysilyl groups at LPSQ-R-Si(OMe)_3_ from undesired hydrolytic degradation.

The ^1^H NMR spectrum of an example reaction mixture (Figure 1) shows a set of broad peaks corresponding to protons in LPSQ-R-Si(OMe)_3_ and sharp peaks (a triplet (CH_3_) and quartet (-OCH_2_-)) that belong to TEOS. The integration matches the molar ratio used in this reaction. The addition of the mercapto-compound to the vinyl groups at the LPSQ backbone proceeded quantitatively, against the Markovnikov rule. The ^13^C NMR spectrum (Figure 2) also proved that the product structure was as expected. The resonance peak corresponding to carbon atoms connected directly to the silsesquioxane chain (-CH_2_- of type a) is broad due to the steric hindrance effect of the LPSQ chain. The carbon resonances in the linker become sharper with increasing distance from the backbone, proving that their segmental freedom was high and their motion was not disturbed by random crosslinking at the Si(OMe)_3_ groups. This effect was also demonstrated in the ^1^H NMR spectrum (Figure 1).

The ^29^Si NMR spectrum (Figure 3) illustrates the presence of two structurally different components in the reaction mixture. The resonances of the silicon atoms in the backbone (T and M type) are much broader than those of Si(OEt)_4_, as well as the resonances of -CH_2_Si(OMe)_3_ in the side substituents grafted to the LPSQ chain. It should be noted that silicon nuclei in the side alkoxysilyl units are represented by a single resonance peak (−38.2 ppm). TEOS is represented by Q-type silicon atoms at −77.5 ppm that are accompanied in the higher field by a small peak (−76.7 ppm, 3.5% of the total Q-unit integration), which was attributed to traces of Si(OEt)_3_(OH). This clearly shows that the proposed modification of LPSQ-Vi by thiol-ene addition in TEOS solution can be successfully applied to modify polysilsesquioxanes (and potentially also polyhedral oligosilsesquioxanes, POSS) with hydrolysis-sensitive functional groups.

### 3.2. Preparation of Hybrid Silica by Sol–Gel Method

Hybrid silicas were prepared following the procedure of the templated sol–gel process under acidic conditions. Polyethylene oxide-polypropylene oxide of a triblock structure (Pluronic^®^ F-127) was used as the structure-directing agent (SDA), or the template. The structure and porosity of silica-based materials can be controlled in sol–gel chemistry by the addition of various surfactants (non-ionic, anionic, or cationic). Triblock water-soluble polymers bearing oxygen-containing functional groups are especially interesting regarding their biomimetic effect in the mechanism of silanols condensation starting from monomer (e.g., Si(OH)_4_) to three-dimensional mesostructured polymers [74]. For example, mesoporous silica (Santa Barbara Amorphous-15 (SBA-15)) was synthesized under acidic conditions with the use of the non-ionic triblock copolymer Pluronic P123 (EO_20_PO_70_EO_20_) [75]. In this case, the formation of hydrogen bonds between the non-ionic surfactant and the silanol groups was favoured. P123 can be removed from the mesostructured silica by solvent extraction with ethanol or by heating at 140 °C for 3 h. Pluronic^®^ F-127 belongs to the same group of SDA, and similar interactions can be expected.

Four groups of experiments (Table 1) differing by the composition of co-monomers (LPSQ-R-Si(OMe)_3_ and TEOS) and the amount of Zn(OAc)_2_ were conducted to estimate the role of specific reaction conditions for the formation of hybrid silica particles in the studied system. Experiments of type I were conducted only with TEOS as a single monomer. It was added dropwise to an aqueous solution of the triblock template, containing HCl and NaCl. In the experiments of type II, Zn(OAc)_2_ was added to the solution of Pluronic^®^ F-127 before the addition of TEOS. A mixture of polymeric LPSQ-R-Si(OMe)_3_ and monomeric TEOS was used in experiments of type III, and their modification was conducted with the addition of Zn(OAc)_2_ (type IV). The silica and hybrid silica that precipitated from the reaction mixtures were purified and dried. After the workup, their morphology was examined with scanning electron microscopy (SEM), thermogravimetric analysis (TGA/DTA), and structural analysis by ^13^C and ^29^Si NMR in the solid state.

#### 3.2.1. The Effect of Sol–Gel Conditions on the Morphology of Silica Particles

**Figure 4:**
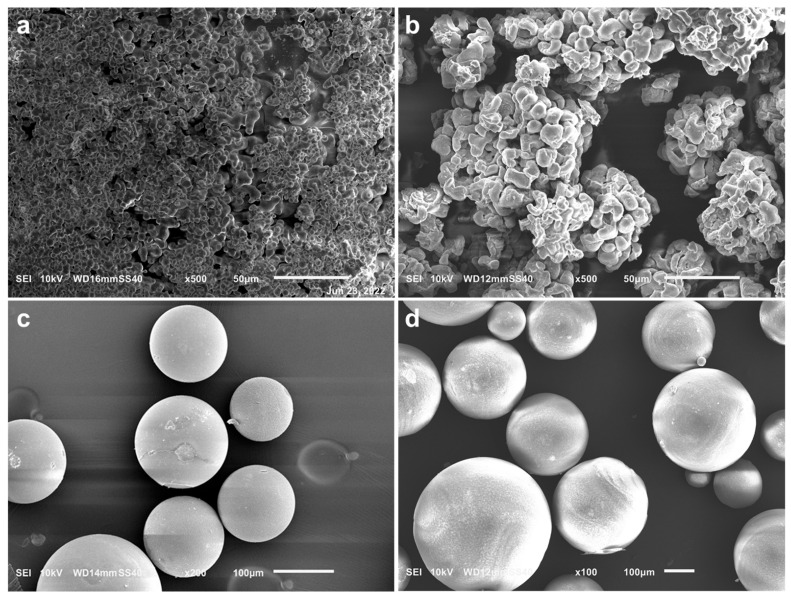
Micrographs of samples I-3 (**a**), II-2 (**b**), III-4 (**c**), and IV-4 (**d**)—the effect of monomer composition on the shape and size of microparticles.

The morphology of the prepared hybrid particles under the applied sol–gel process conditions was assessed by scanning electron microscopy (SEM). The micrographs showed that the microparticles of silica obtained in experiments type I and II had an irregular shape (Figure 4a,c). Interestingly, the hybrid silica microparticles obtained using LPSQ-R-Si(OMe)_3_ in type III and IV experiments were spherical.

**Scheme 2:**
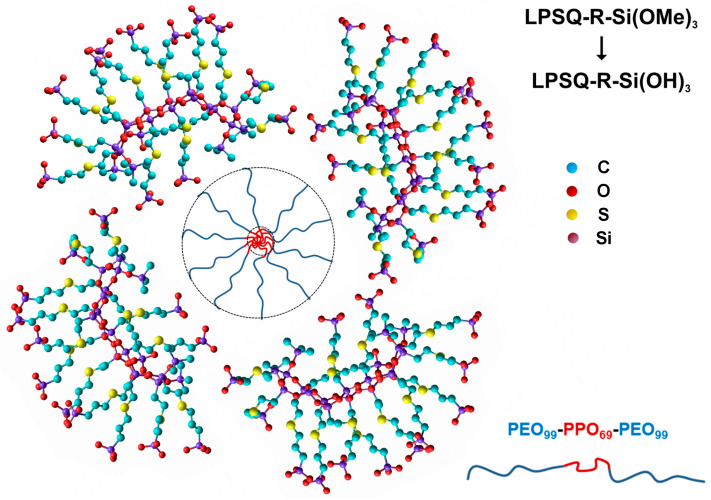
Postulated interactions between oligomeric LPSQ-R-Si(OH)_3_ and micelles of Pluronic^®^ F-127. Color of elements in the structure of LPSQ-R-Si(OH)_3_ as in the legend (hydrogen atoms were omitted for clarity). Simplified image of a micelle formed by the triblock polymeric surfactant is in the middle of the scheme.

The postulated mechanism of the formation of spherical core-shell particles is shown in Scheme 2. Pluronic^®^ F-127 is a triblock copolymer known to form supramolecular structures (including spherical micelles) depending on the concentration, temperature, polarity, and pH of the medium [76,77,78]. In aqueous solutions, micelles formed by this surfactant feature a hydrophobic core composed of a PPO block surrounded by a hydrophilic corona containing PEO chains. Hydrolysis of LPSQ-R-Si(OMe)_3_ yields oligomers with reactive silanol groups of high affinity to PEO (Scheme 2). The presence of a ladder-like backbone makes their coiling difficult (compared to their linear polysiloxane analogues). However, the modeled structure of LPSQ-R-Si(OH)_3_ suggests that the silsesquioxane backbone does not resemble an ideally rigid rod. Such silsesquioxane oligomers can surround the hydrophilic corona of the micelle.

**Figure 5:**
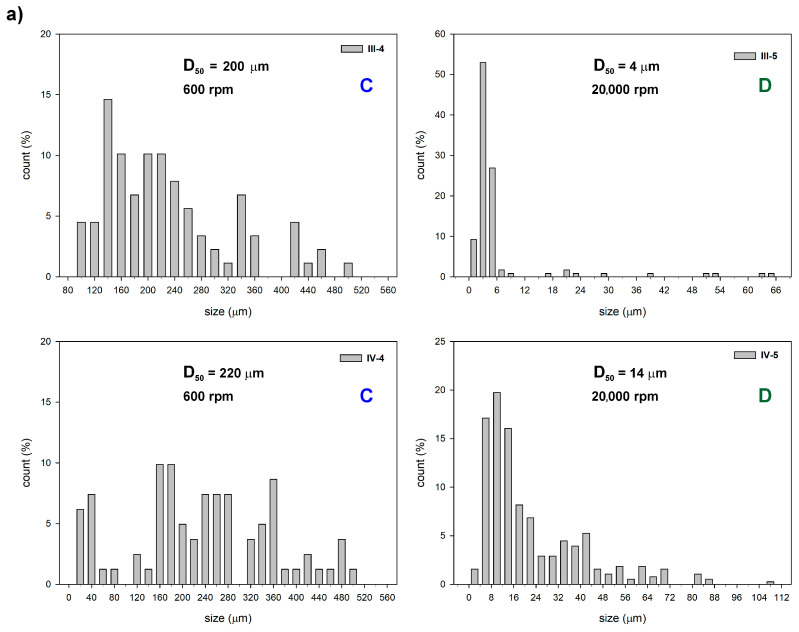

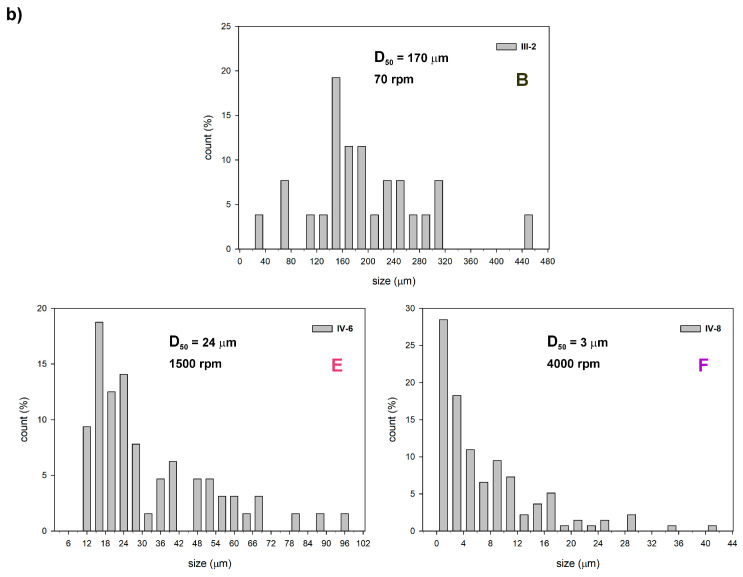
Distribution of spherical particles depending on the reaction conditions (different stirring rates (B–F—type of stirring mode (see Section 2.2.2) and ratio [-Si(OMe)_3_]_0_/[TEOS]_0_ = 0.97 (**a**) or = 1.95 (**b**)).

Comparative analysis showed that regardless of the alkoxysilane monomer/macromonomer used, particle size increased in experiments conducted with the addition of Zn(OAc)_2_. Figure 5 shows the particle-size distribution (DPS) obtained for samples (type III and IV) of different compositions produced with different mixing modes. The particle size (diameter) data were grouped into intervals, and the count (%) corresponds to the percentage of particles in each interval. The rate of stirring, the ratio [TEOS]_0_/[LPSQ-R-Si(OMe)_3_]_0,_ and pH of the reaction mixture were crucial for the size and DPS of the microparticles.

The rate of hydrolysis and condensation reactions in the sol–gel process is affected by steric and inductive factors, but it is the pH dependence of the hydrolysis, condensation, and dissolution reactions that governs the structural features of the formed macromolecules [79,80]. Under acidic conditions, the hydrolysis is favoured. It becomes negligible on approaching neutral pH. The rate of hydrolysis is higher than the rate of condensation, and, usually, oligomers with a less branched structure are formed because the protonated silanol preferentially condensates with the least acidic silanol end groups. The minimum of the silanols condensation rate is observed at about pH = 2. However, both under strongly acidic (pH < 1) but at pH > 4, the rate of polycondensation significantly increases. When the reaction medium is alkaline, then the deprotonated silanol attacks the more acidic silanol groups, leading to branching and formation of condensed clusters.

We have found that the pH value of the 0.5 M aqueous solution of HCl containing NaCl and F-127 was equal to 0.45. A decrease in the acidity of the reaction medium on addition of Zn(OAc)_2_ was noted (for example, pH in experiments II-2 and II-1 was equal to 0.89 and 3.30, respectively). The observed higher values of pH with increasing amount of Zn(OAc)_2_ added to the HCl_aq_/NaCl solution indicate that the hydrolysis of ZnCl_2_ (formed in situ) yielded water-soluble compounds xZn(OH)_2_·yZnCl_2_·zH_2_O that are more basic than ZnCl_2_. The change was significant enough to cause a shift in the relative equilibria of the reactions of hydrolysis of alkoxysilanes and the condensation of silanols. Due to the enhanced polycondensation rate, the experiments of type II and IV (with Zn(OAc)_2_ added to the reaction medium) yielded particles of larger size (a relative increase of the SiO_4/2_ fraction derived from TEOS molecules). These studies also helped to clarify other observations (a relative increase of the Q units in the hybrid silica of type IV and its higher thermal stability—a detailed discussion is presented in Section 3.2.2 and Section 3.2.3).

**Figure 6:**
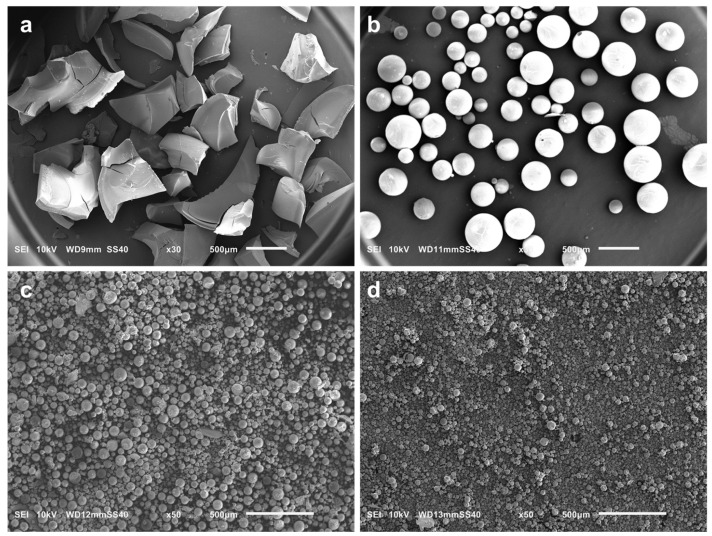
Micrographs of samples IV-2 (**a**), IV-4 (**b**), IV-5 (**c**), and IV-8 (**d**)—the effect of stirring on the shape and size of microparticles.

Samples III-4 and IV-4 were prepared with stirring type C. The particles in both samples were large, and their DPS was quite wide. The average microsphere size in III-4 (median particle size D_50_ = 200 µm) is slightly smaller than that in IV-4 (D_50_ = 220 µm). Micrographs of type IV samples prepared under different stirring modes are shown in Figure 6. As expected, faster stirring resulted in the formation of much smaller particles. Their DPS was also narrow (Figure 5). Interestingly, the average size of IV-8 (F) was smaller (D_50_ = 3 µm) than IV-5 (D) (D_50_ = 14 µm), but the polydispersity was similar. The F stirring mode was fast but short. It is possible that some of the initially formed microcapsules merged when the stirring was stopped, although the spherical particles were stable enough to maintain their shape during the entire processing.

These observations point to an efficient crosslinking of silanol groups in the hydrolyzed LPSQ-R-Si(OMe)_3_ at the beginning of the sol–gel process. The proximity of -R-Si(OH)_3_ formed in situ facilitated polycondensation. Even sample IV-2, which was obtained at prolonged but relatively slow stirring (type B), contained crushed pieces (Figure 6a) of shape and curvature characteristic of large, spherical objects with a hard shell and internal void. Such large spheres can also crack during the drying process due to the internal stress generated in the thick walls.

**Figure 7:**
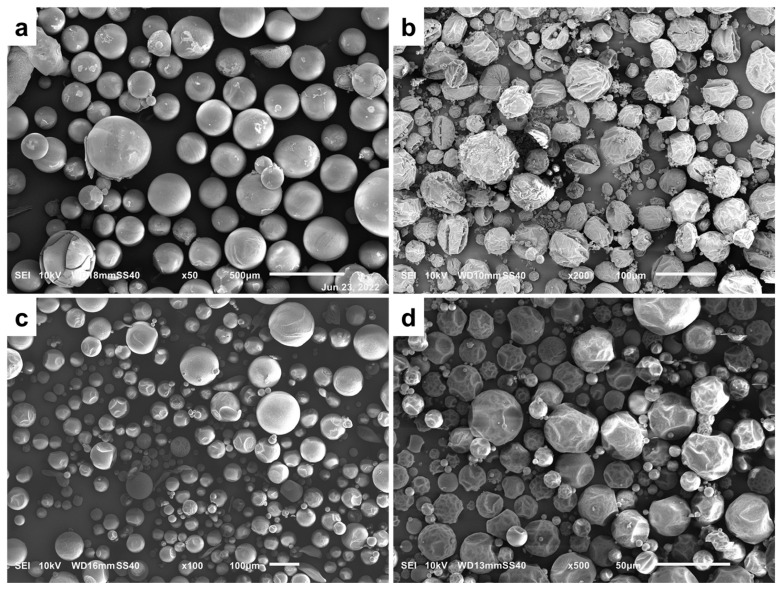
Micrographs of samples III-2 (**a**), III-5 (**b**), IV-6 (**c**), and IV-8 (**d**)—the effect of the concentration of TEOS, the pH of the medium, and the rate of stirring on the shape and size of microparticles.

**Figure 8:**
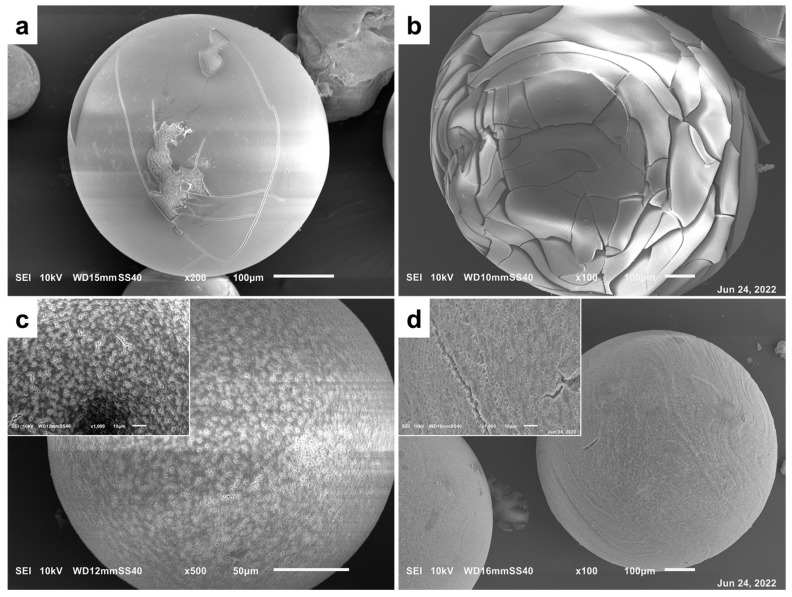
Micrographs of samples III-4 (**a**,**b**) and IV-4 (**c**,**d**) before (**a**,**c**) and after pyrolysis (**b**,**d**)—the effect of the increase of the pH of the reaction medium by addition of Zn(OAc)_2_ on the thermal stability of microparticles (insets in Figure 8c,d show magnifications of ×1000).

The polydispersity of particles III-5 obtained with fast stirring (D) was high, although the D_50_ ≈ was 4 µm. Their shell was apparently thinner and more prone to collapse during drying than that of III-2 particles obtained with a lower amount of TEOS but with slower stirring (B, D_50_ ≈ 170 µm) (Figure 7a,b). The addition of Zn(OAc)_2_ to the reaction mixture increased its pH and improved the condensation efficiency of silanols, yielding microparticles with higher wall compactness despite reduced amounts of TEOS (Figure 7c,d). It also had a significant effect on the structural stability of the hybrid silica particles during pyrolysis. The surface of microspheres III-4 cracked severely (Figure 8b), while those of IV-4 remained almost unchanged (Figure 8d).

**Figure 9:**
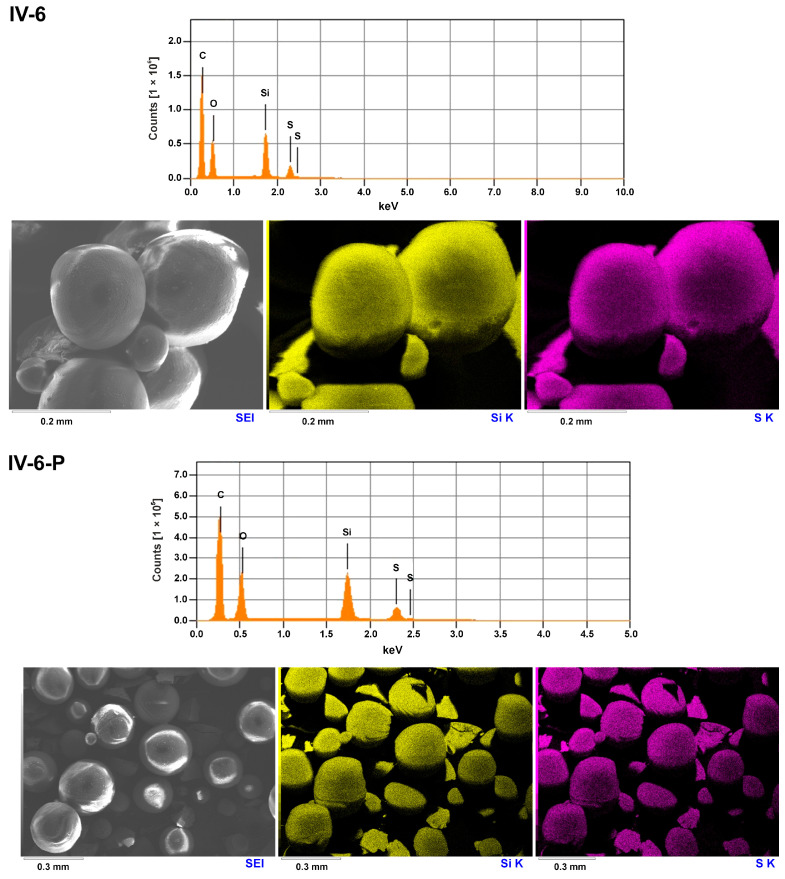
SEM-based EDS spectra and secondary electron images (SEI) of sample IV-6 before and after the pyrolysis (IV-6-P) with EDS mapping of silicon (yellow) and sulfur (pink).

The elemental composition of selected representative samples (II-1, IV-4, and IV-6, before and after the pyrolysis) was studied with scanning electron microscopy combined with energy dispersive X-ray spectroscopy (SEM-EDS). The method combines high-resolution surface imaging with elemental analysis. The results indicated that the materials obtained in experiments conducted under pH = 3.3, with Zn(OAc)_2_ added to the solution, did not contain zinc atoms (at least in the amounts detectable by SEM-EDS (Figure 9, Figures S1 and S2). It was found that silicon and sulfur atoms were evenly distributed in the studied samples of hybrid silica IV. It means that the structure of microspheres is uniform, and they are not built of separate agglomerates originating from LPSQ-R-Si(OMe)_3_ and TEOS.

The structure of these samples was also studied with wide-angle X-ray scattering (WAXS). X-ray diffraction techniques allow for the identification of different crystalline phases characteristic of zinc derivatives (such as ZnCl_2_ and xZn(OH)_2_·yZnCl_2_·zH_2_O) if they were present in these samples [81]. The diffractograms (Figure S3) showed that silica of types II and IV did not contain any crystalline components.

#### 3.2.2. Thermal Stability of the Hybrid Silica

Silica prepared with the use of Pluronic^®^ F-127 is usually subjected to high-temperature pyrolysis to remove the template [18,19,20]. We performed a thermogravimetric analysis of this surfactant to find the most suitable conditions for its removal from hybrid silica. The complete thermal decomposition of the surfactant occurred at 200 °C (in air) and 350 °C (in N_2_ atmosphere) (Figure S4). Thermograms of both silica type I (Figure S5a,b) and type II (Figure S6a,b) featured broad peaks with maximum degradation temperature (T_max_), which corresponded to the T_max_ of Pluronic^®^ F-127 heated under the same conditions. After pyrolysis at 217 °C in air (2 h), the thermograms showed no signs of surfactant decomposition (Figures S5c,d and S6c,d). The decrease in weight of the samples in the range of 20–100 °C can be attributed to thermal desorption of H_2_O.

**Figure 10:**
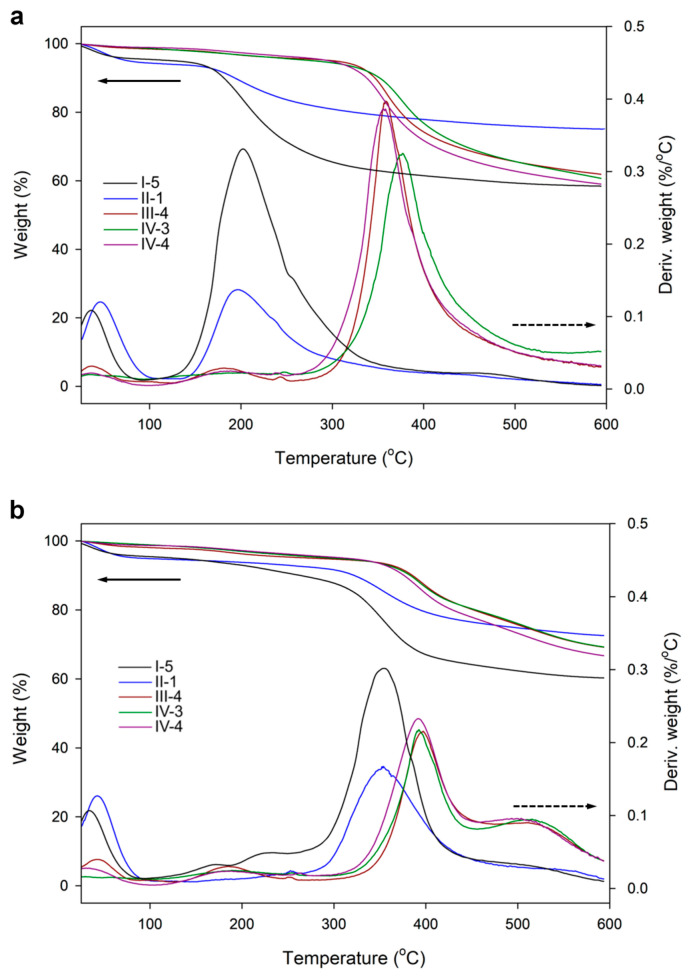
Comparative thermogravimetric analysis of hybrid silicas (10 °C/min ramp) in air (**a**) or in N_2_ atmosphere (**b**) (solid black arrow—TGA, dashed black arrow—DTA).

**Figure 11:**
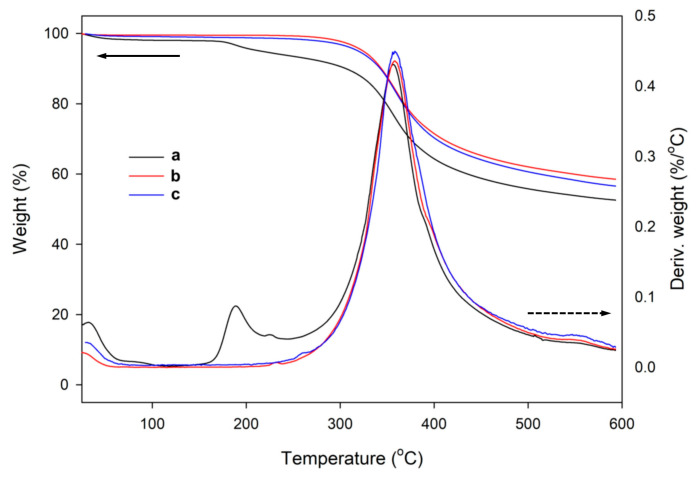
TGA traces (10 °C/min, air atmosphere) registered for sample III-1 as prepared (a), after the isothermal treatment (b) and pyrolyzed for 2 h at 217 °C in a furnace (c) (solid black arrow—TGA, dashed black arrow—DTA).

The comparison of thermogravimetric traces of those samples and the materials obtained using LPSQ-R-Si(OMe)_3_ showed a significant difference in their thermal behavior (Figure 10). The thermal decomposition of hybrid silica types III and IV, conducted in air and in N_2,_ is very different from those of SiO_2_ types I and II. Only a small mass loss was recorded at 20–100 °C and at 200 °C in air (H_2_O desorption and Pluronic^®^ F-127 pyrolysis). The decomposition traces in N_2_ did not contain a peak that could indicate surfactant degradation. The small amount of adsorbed water can be explained by the hydrophobicity of the hybrid silica due to the presence of an incorporated organic linker. The decomposition of organic structures occurs at much higher temperatures (350 °C in air and 400 °C in N_2_, regardless of the pH and the content of TEOS used for the synthesis). These temperatures are characteristic of Si-C bond cleavage in hybrid silsesquioxane polymers, as shown by our previous studies of the thermal decomposition of similar systems in oxidative and inert atmospheres by TGA, FTIR, and GC-MS [82]. The absence of the characteristic Pluronic^®^ F-127 decomposition peak indicates that, in contrast to SiO_2_ prepared under the same conditions, the hybrid silica did not contain large amounts of the template. This explanation is supported by the fact that only the sample III-1 prepared with a larger amount of Pluronic^®^ F-127 featured the characteristic weight loss at about 200 °C (Figure 11).

**Figure 12:**
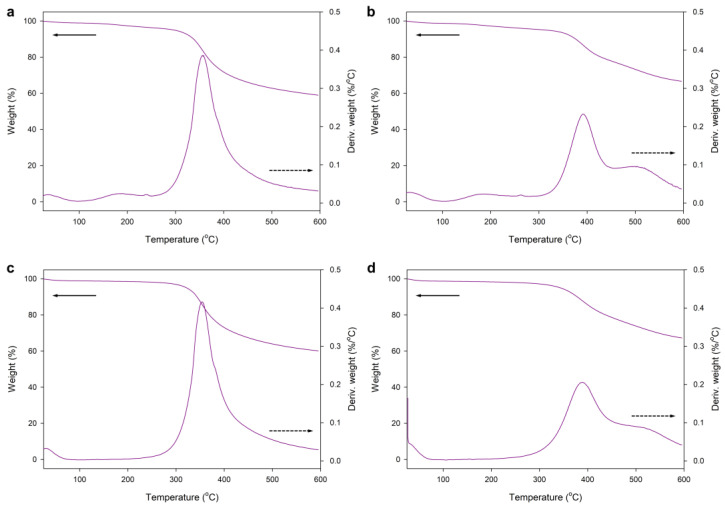
TGA traces (10 °C/min) of sample IV-4 as prepared (**a**,**b**) and after pyrolysis for 2 h at 217 °C in a furnace (**c**,**d**). Analysis in air (**a**,**c**) or N_2_ (**b**,**d**) atmosphere (solid black arrow—TGA, dashed black arrow—DTA).

TGA/DTA traces of sample IV-4 (as prepared and after pyrolysis for 2 h at 217 °C in the oven) (Figure 12) indicated that the amount of the template was low. After pyrolysis, the characteristic peak disappeared, and a mass loss occurred at T > 300 °C. This result proved the high thermal stability of the incorporated organic fraction (originating from the spacer connecting the silsesquioxane ladder skeleton and the silica network formed during the sol-gel process). The similarity of the weight decrease in air and N_2_ atmosphere (about 30% at T_max_) also points to the high oxidative stability of the hybrid silica. To confirm the postulated path of thermal decomposition, we also investigated the changes in the structure of the prepared silicas before and after pyrolysis (Section 3.2.3).

#### 3.2.3. Studies on the Chemical Structure of Hybrid Silica with Solid State NMR Spectroscopy

Structural studies using solid-state NMR spectroscopy (^13^C and ^29^Si nuclei) were performed to investigate the differences in the molecular morphology of the hybrid silica depending on the composition of co-monomers used in the sol–gel reaction, the influence of pH, and structural changes induced by heat treatment of the hybrid products.

**Figure 13:**
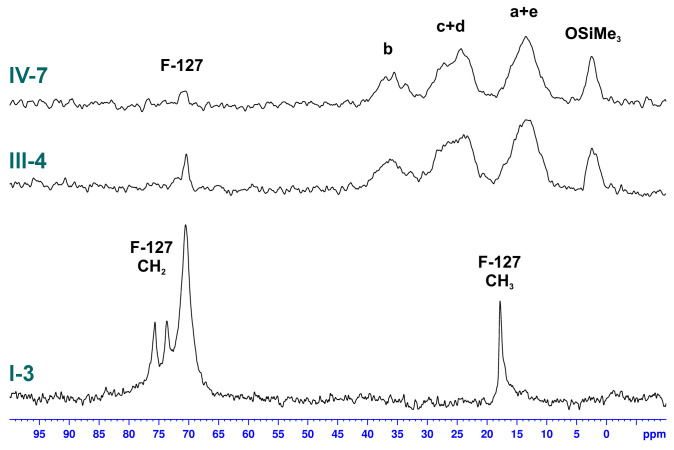
^13^C SS NMR spectra (HP Dec) of samples I-3, III-4 and IV-7 (before pyrolysis). Symbols a–e indicate different types of methylene protons in LPSQ-R-Si(OMe)_3_ (as in Figure 2).

The comparison of ^13^C SS spectra (Figure 13) of the materials obtained with different co-monomers and not pyrolyzed illustrates two characteristic features. The silica prepared with LPSQ-R-Si(OMe)_3_ contained a rather small amount of Pluronic^®^ F-127 (characteristic resonances at 17, 70, 73, and 76 ppm). Although a direct comparison is not possible due to the lack of characteristic ^13^C NMR signals for the silica obtained with TEOS, the content of the surfactant in the exemplary spectra of samples III-4 and IV-7 is much lower than could be expected based on the relative concentration of the reagents. The samples of hybrid materials prepared from LPSQ-R-Si(OMe)_3_ featured four broad resonances that correlate with the spectrum of native LPSQ-R-Si(OMe)_3_ (Figure 2). The peaks around 3 ppm, 14 ppm, 24 ppm, and 36 ppm were assigned to carbon atoms in the -OSiMe_3_ chain-end groups and methylene units in the side chains (a + e, c + d, and b), respectively. The presence of the end groups proves the stability of the LPSQ framework under the applied conditions. The increase of pH of the reaction medium caused by the addition of Zn(OAc)_2_ did not affect this trend.

**Figure 14:**
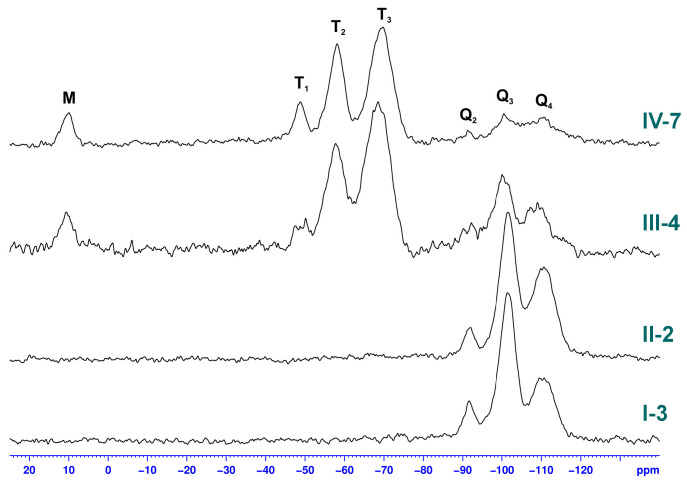
^29^Si SS NMR spectra (HP Dec) of samples I-3, II-2, III-4, and IV-7 (before pyrolysis).

However, the influence of the increased pH and more effective condensation of silanols derived from both co-monomers is visible in ^29^Si SS NMR spectra (Figure 14). The spectrum of silica obtained with TEOS is characterized by only three characteristic peaks, which correspond to Si atoms connected with four oxygens in different chemical environments: −91 ppm (Q_2_: Si(OSi-)_2_(OH)_2_), −101 ppm (Q_3_: Si(OSi-)_3_(OH)_1_), and −112 ppm (Q_4_: Si(OSi-)_4_). The majority of silicon atoms with Q_3_ characteristics indicate that the amount of silanol groups in this silica is significant. At higher pH, a significant increase of Q_4_ units can be observed, suggesting that although not all SiOH are converted to siloxane bonds, the structure of hybrid II-2 is more stable than that of I-3.

The change of pH also had an effect on the structure of hybrid silicas. The resonance peaks in the ^29^Si SS NMR spectrum of sample IV-7 appear to be more defined than those of III-4. However, the influence of pH on the relative ratio of T-structured silicon atoms that form both the LPSQ backbone and the cross-linked matrix formed during the sol–gel process (−51 ppm (T_1_: -CH_2_Si(OSi-)_1_(OH)_2_), −58 ppm (T_2_: -CH_2_Si(OSi-)_2_(OH)_1_), and −68 ppm (T_3_: -CH_2_Si(OSi-)_3_) cannot be directly estimated. The T_3_ units correspond to the silicon atoms in the LPSQ backbone but may also be formed during the cross-linking. Although the ratio between T_2_ and T_1_, which originate from -Si(OMe)_3_, is similar, these resonance peaks seem to be more defined in IV-7. Together with the higher Q_4_/Q_3_ ratio in IV-7, this indicates a role of the increased pH in the formation of siloxane bonds in the sol–gel process. The presence of M units (-OSiMe_3_ groups) at 10 ppm proves the structural robustness of LPSQ under the reaction conditions used.

**Figure 15:**
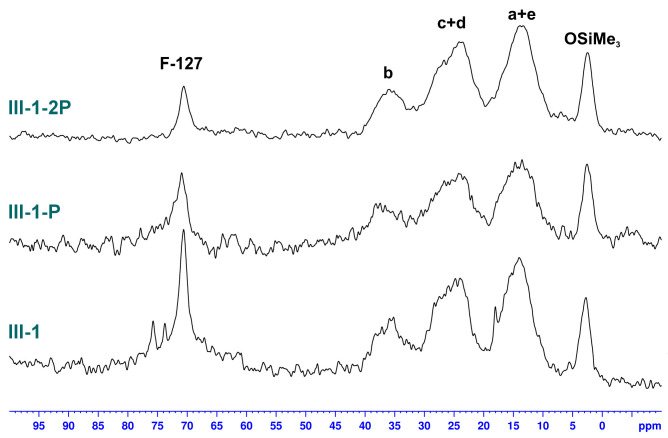
^13^C SS NMR spectra (HP Dec) of samples III-1 before (III-1) and after the first (III-1-P) and second (III-1-2P) thermal treatment. Symbols a–e indicate different types of methylene protons in LPSQ-R-Si(OMe)_3_ (as in Figure 2).

**Figure 16:**
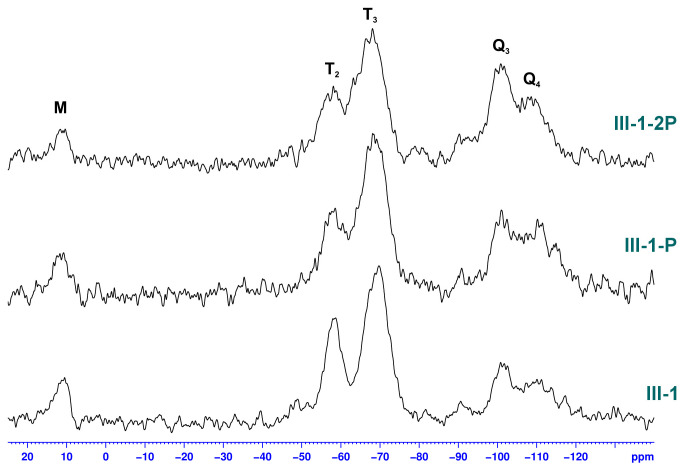
^29^Si SS NMR spectra (HP Dec) of samples III-1 before (III-1) and after the first (III-1-P) and second (III-1-2P) thermal treatment.

Studies on the effect of heat treatment on the morphology of the hybrid silica were also conducted. The solid-state NMR spectra of the exemplary product III-1, which was obtained with LPSQ-R-Si(OMe)_3_ and TEOS, are shown in Figure 15 and Figure 16. This sample was obtained with a larger amount of Pluronic^®^ F-127 and magnetic stirring during the sol–gel reaction. Both factors influenced the amount of residual triblock surfactant in the sample (significantly higher than in the other products). Pyrolysis of III-1 caused a decrease in the characteristic resonance at 70 ppm but did not completely remove the template despite repeated heat treatment. More importantly, both thermal treatments did not cause any change in the pattern of resonance signals belonging to LPSQ (methylene groups in the side chains and -OSiMe_3_ end groups). This proves the high thermal stability of the organic component in the cross-linked hybrid silica. Nevertheless, some redistribution of siloxane bonds can be observed in ^29^Si solid-state NMR spectra (Figure 16). The sample before the thermal work-up featured silicon atoms of M, T_2_, T_3_, Q_3,_ and Q_4_ types. While M and Q units remained almost unchanged, the ratio between T_3_ and T_2_ increased, which proves the condensation of some silanol groups. Both peaks become broader, which illustrates the redistribution of siloxane bonds in the hybrid silica after thermal cross-linking.

The T/Q ratio in the ^29^Si NMR spectra is apparently larger than the molar ratio of -Si(OMe)_3_ groups to TEOS in the mixture of reagents obtained in the first step (Table 1). Although the peak broadening might somewhat affect the result, the effect can be attributed to the preferential polycondensation of the side groups grafted onto LPSQ and less effective incorporation of oligomers formed from TEOS.

Comparative analysis of ^13^C SS NMR spectra showed that both the TEOS concentration in the reaction mixture and the stirring rate affect the content of template in the final product (before pyrolysis) (Figures S4 and S5). Reducing the amount of TEOS resulted in a decrease in the wall thickness of the hybrid microcapsules, which facilitated the removal of residual Pluronic^®^ F-127. On the other hand, the template-to-reagent ratio apparently had no effect on its residual concentration. ^13^C NMR and ^29^Si solid state spectra of the hybrid silica (Figures S6 and S7) are similar regardless of the TEOS concentration. As expected, the only difference is the amount of Q units in the ^29^Si NMR spectra. The ratio of T_1_, T_2,_ and T_3_ is the same irrespective of the content of TEOS. This suggests that polycondensation between the pendant silanol groups of hydrolyzed LPSQ-R-Si(OMe)_3_ takes place preferentially prior to hetero-condensation with Si(OH)_4_ and oligomers derived from TEOS.

Samples IV-3 and IV-4, subjected to pyrolysis, had reduced content of silicon atoms T_1_ and T_2_ (Figures S6 and S7). At the same time, the peaks corresponding to silicon atoms T_3_ and Q_3_ increased, which indicates that the hetero-condensation took place mainly in the later stages of the process. Interestingly, no broadening of the ^29^Si resonance peaks was observed to the extent reported for sample III-1 (Figure 16). Moreover, the signals are better resolved in the pyrolyzed sample IV-3-P than in IV-4-P obtained with a higher amount of TEOS. This effect can be attributed to the reinforcement of the hybrid silica due to a higher extent of silanol group condensation in samples of series IV.

## 4. Conclusions

Spherical hybrid silica particles were obtained in good yields when linear multialkoxyfunctional macromolecules–polysilsesquioxanes (LPSQ) functionalized with trimethoxysilyl groups in the side chains (LPSQ-R-Si(OMe)_3_)– were used together with TEOS as comonomers in a sol–gel reaction conducted under acidic conditions in the presence of Pluronic^®^ F-127 as a template. Precipitation of the hybrid silica microspheres was much faster than silica derived from TEOS alone, and we demonstrated that the specific structure of LPSQ-R-Si(OMe)_3_ was crucial for this effect. Structural studies with ^13^C and ^29^Si NMR in the solid state proved the preferential polycondensation of silanols derived from LPSQ-R-Si(OMe)_3_, as well as the robustness of the LPSQ backbone under the applied conditions. In contrast to silica obtained exclusively from TEOS, the as-prepared hybrid microspheres did not contain large amounts of residual template. Moreover, the hybrid materials were hydrophobic and exhibited high thermal and oxidative stability of the organic fraction. The size of microspheres can be controlled by the stirring rate and by the pH of the reaction mixture. The latter can be modulated by the addition of zinc acetate to the sol–gel reaction. The induced increase of pH resulted in the structural enhancement of the hybrid microparticles due to the increased degree of condensation of the silanol groups and higher amounts of Q units (more efficient hetero-condensation with Si(OH)_4_ derived from TEOS).

It should be noted that although the proposed method of LPSQ functionalization (photo-initiated thiol-ene addition conducted in TEOS) was effective and innovative, it would be very important to limit the amount of TEOS at this step. The addition of Zn(OAc)_2_ allowed pH modulation, but the microspheres did not contain any Zn^2+^ compounds that could be converted to ZnO during thermal work-up. This disadvantage can be eliminated by carefully adjusting the pH at various stages of the sol–gel process. Any future work should be targeted in this direction, as this could potentially lead to fine dispersion of ZnO nanocrystals in the silica matrix of the microspheres.

The approach presented in this work demonstrates the potential of the alkoxy-functional all-organosilicon macromolecular monomers in the synthesis and properties of hybrid materials. The proposed method can be translated to other organosilicon polymers and oligomers that can be employed to produce hollow silica particles. Future research should focus on using less complex macromonomers (instead of LPSQs) and reducing particle size (towards the nanoscale), followed by exploring the use of the hybrid particles as nanocarriers for active ingredients.

## Figures and Tables

**Table 1:** Concentration of reagents and parameters applied during the synthesis of hybrid silica.

Sample	-Si(OMe)_3_ [mmol]	TEOS [mmol]	Zn(OAc)_2_ [mmol]	F-127 [g]	X	Y [%]	W [wt%]	P [%]
I-1	-	8.82	-	0.92	A	50.9	63.48	18.97
I-2	-	8.82	-	0.41	B	95.0	43.65	16.2
I-3	-	8.82	-	0.41	C	105.3	43.65	24.31
I-4	-	4.48	-	0.41	D	49.3	60.41	23.02
I-5	-	8.82	-	0.41	D	94.5	43.65	17.49
II-1	-	8.82	4.56	0.41	B	59.4	29.65	14.09
II-2	-	8.82	2.32	0.41	E	103.1	35.18	23.87
II-3	-	8.82	2.32	0.41	F	39.0	35.18	12.16
III-1	4.36	4.48	-	0.92	A	47.8	44.35	6.04
III-2	4.36	2.24	-	0.41	B	68.7	28.67	4.05
III-3	4.36	4.48	-	0.41	B	72.3	26.21	5.34
III-4	4.36	4.48	-	0.41	C	64.0	26.21	3.81
III-5	4.36	4.48	-	0.41	D	81.5	26.21	2.54
IV-1	4.36	2.24	4.56	0.41	B	56.7	21.88	4.66
IV-2	4.36	4.48	4.56	0.41	B	100.6	20.42	4.42
IV-3	4.36	2.24	4.56	0.41	C	37.4	21.88	2.07
IV-4	4.36	4.48	4.56	0.41	C	34.1	20.42	3.15
IV-5	4.36	4.48	4.56	0.41	D	52.7	20.42	6.46
IV-6	4.36	2.24	4.51	0.36	E	44.8	19.79	4.56
IV-7	4.36	4.48	5.10	0.41	E	34.7	19.89	4.78
IV-8	4.36	2.24	4.51	0.36	F	85.2	19.79	3.02
IV-9	4.36	4.48	5.10	0.41	F	55.1	19.89	3.55

X—stirring mode (see Section 2.2.2). Y—solid product yield. W—theoretical weight content of Pluronic F-127 in the crude mixture of solid products. P—average sample weight decreases after the pyrolysis.

## Data Availability

The original contributions presented in this study are included in the article/Supplementary Materials. Further inquiries can be directed to the corresponding author.

## Supplementary Materials

The following supporting information can be downloaded at: https://www.mdpi.com/article/10.3390/ma18143357/s1, Figure S1: SEM-based EDS spectra and secondary electron images (SEI) of sample II-1 before and after the pyrolysis (II-1-P) with EDS mapping of silicon (yellow); Figure S2: SEM-based EDS spectrum and SEI of sample IV-4-P with silicon (yellow) and sulfur (pink) EDS mapping; Figure S3: Wide-angle X-ray scattering (WAXS) diffractograms of samples II-1 and IV-4; Figure S4: TGA and DTA traces registered for pure Pluronic^®^ F-127 in air (a) and N_2_ atmosphere (b) (heating ramp 10 °C/min) (solid black arrow—TGA, dashed black arrow—DTA); Figure S5: TGA and DTA traces (10 °C/min) of sample I-5 B-33/14 as prepared (a+b) and after pyrolysis for 2 h at 217 °C in a furnace (c+d). Analysis in air (a+c) or N_2_ (b+d) atmosphere (solid black arrow—TGA, dashed black arrow—DTA); Figure S6: TGA and DTA traces (10 °C/min) of sample II-1 as prepared (a+b) and after pyrolysis for 2 h at 217 °C in a furnace (c+d). Analysis in air (a+c) or N_2_ (b+d) atmosphere (solid black arrow—TGA, dashed black arrow—DTA); Figure S7: ^13^C SS NMR spectra (HP Dec) of samples IV-4 and IV-3 before and after the thermal treatment (IV-4-P and IV-3-P, respectively). Symbols a-e indicate different types of methylene protons in LPSQ-R-Si(OMe)_3_ (as in Figure 2); Figure S8: ^13^C SS NMR spectra (HP Dec) of samples IV-7 and IV-6. Symbols a-e indicate different types of methylene protons in LPSQ-R-Si(OMe)_3_ (as in Figure 2); Figure S9: ^29^Si SS NMR spectra (HP Dec) of samples IV-4 and IV-3 before and after the thermal treatment (IV-4-P and IV-3-P, respectively); Figure S10: ^29^Si SS NMR spectra (HP Dec) of samples IV-7 and IV-6.

## Abbreviations

The following abbreviations are used in this manuscript:

LPSQ	Linear polysilsesquioxanes
POSS	Polyhedral oligomeric silsesquioxanes
TEOS	Tetraethoxysilane
DMPA	2,2-dimethoxy-2-phenylacetophenone
LPSQ-Vi	Linear poly(vinylsilsesquioxanes)
LPSQ-R-Si(OMe)_3_	Linear polysilsesquioxanes functionalized with trimethoxysilyl groups
Zn(OAc)_2_	Zinc acetate dihydrate
SS NMR	Solid-state nuclear magnetic resonance spectroscopy
HP Dec	High-power decoupling
TGA	Thermogravimetric analysis
DTA	Differential thermal analysis
T_max_	Maximum degradation temperature
SEM	Scanning electron microscopy
DPS	Particle-size distribution
D_50_	Median particle size
SEM-EDS	Scanning electron microscopy equipped with energy dispersive spectroscopy
SEI	Secondary electron image
WAXS	Wide-angle X-ray scattering
