# Supplementary material for: Formation of Hybrid Spherical Silica Particles Using a Novel Alkoxy-Functional Polysilsesquioxane Macromonomer as a Precursor in an Acid-Catalyzed Sol-Gel Process
Source: Materials (Basel). 2025 Jul 17;18(14):3357. doi: 10.3390/ma18143357 (PMC12298626; doi:10.3390/ma18143357)
Supplement: Supplementary file 1 [file materials-18-03357-s001.zip › materials-3702987-supplementary.pdf]

Formation of hybrid spherical silica particles using a novel alkoxy-functional polysilsesquioxane macromonomer as a precursor in an acid-catalyzed sol-gel process.

Suplementarny Information

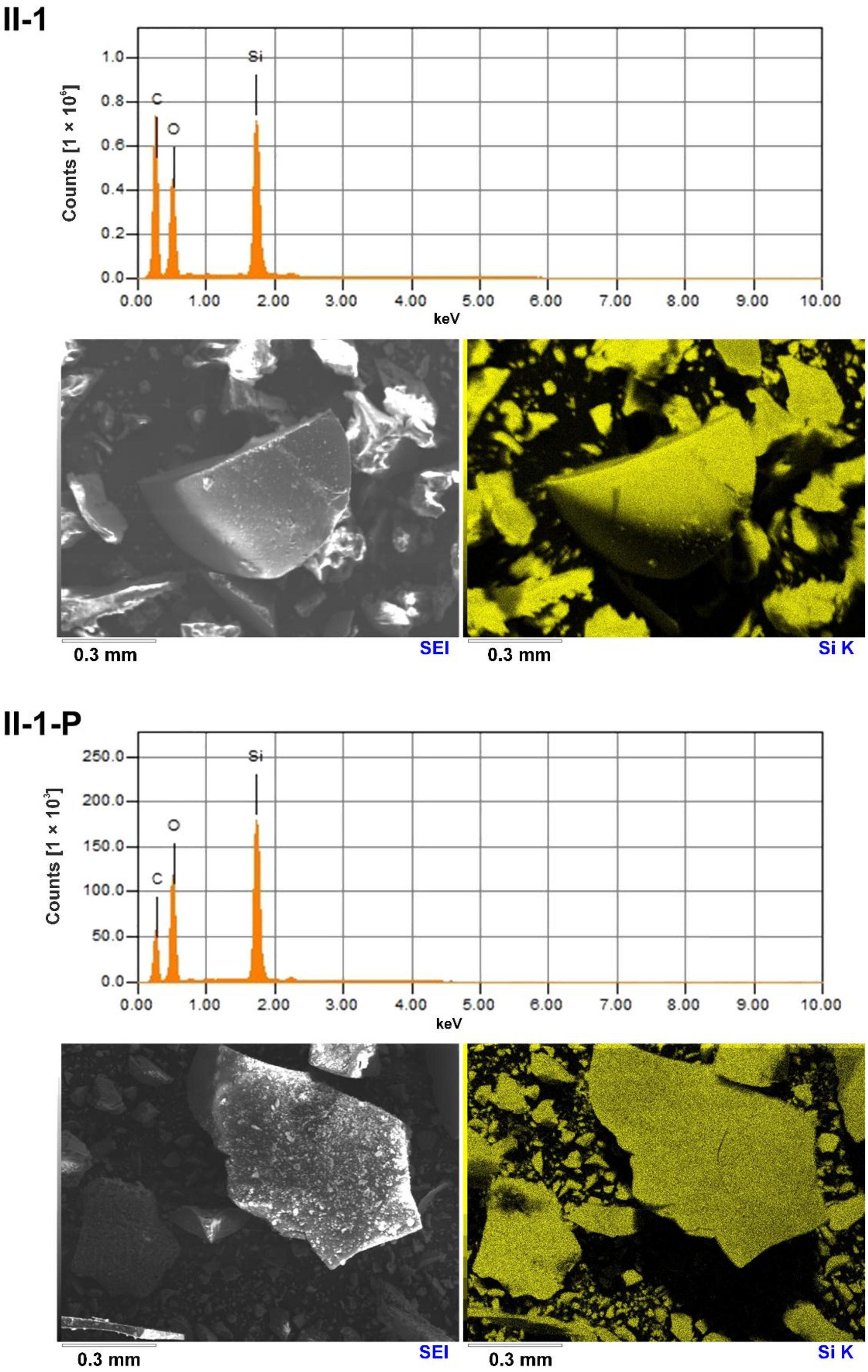

Figure S1. SEM-based EDS spectra and secondary electron images (SEI) of sample II-1 before and after the pyrolysis (II-1-P) with EDS mapping of silicon (yellow).

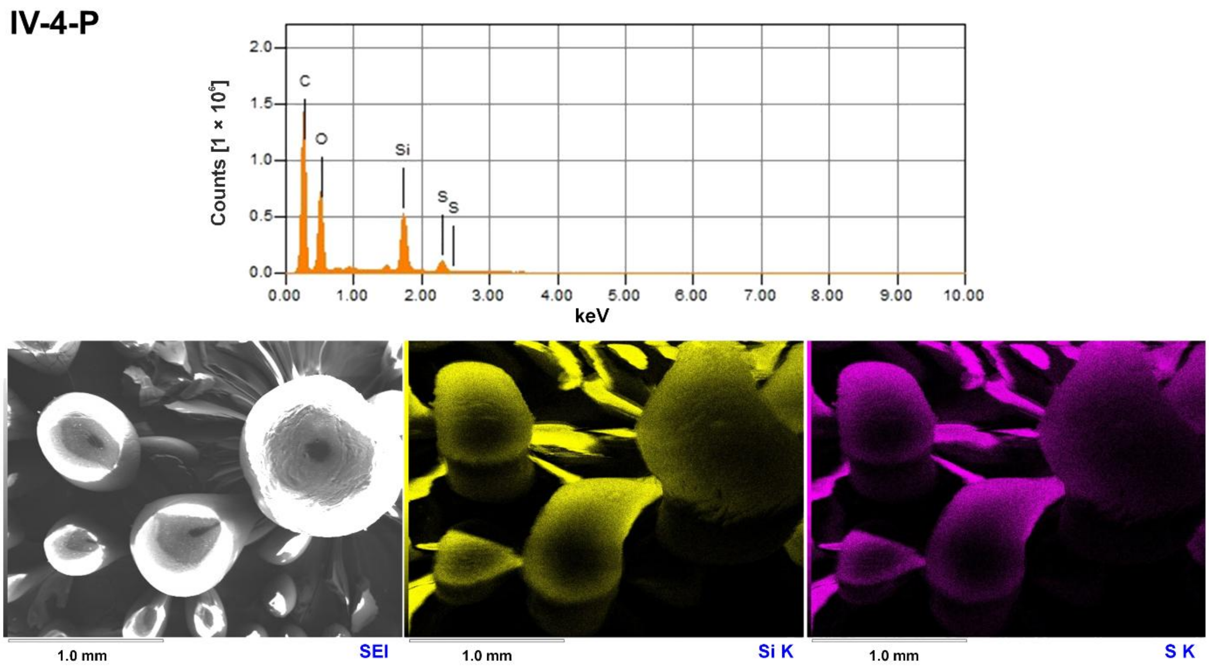

Figure S2. SEM-based EDS spectrum and SEI of sample IV-4-P with silicon (yellow) and sulfur (pink) EDS mapping.

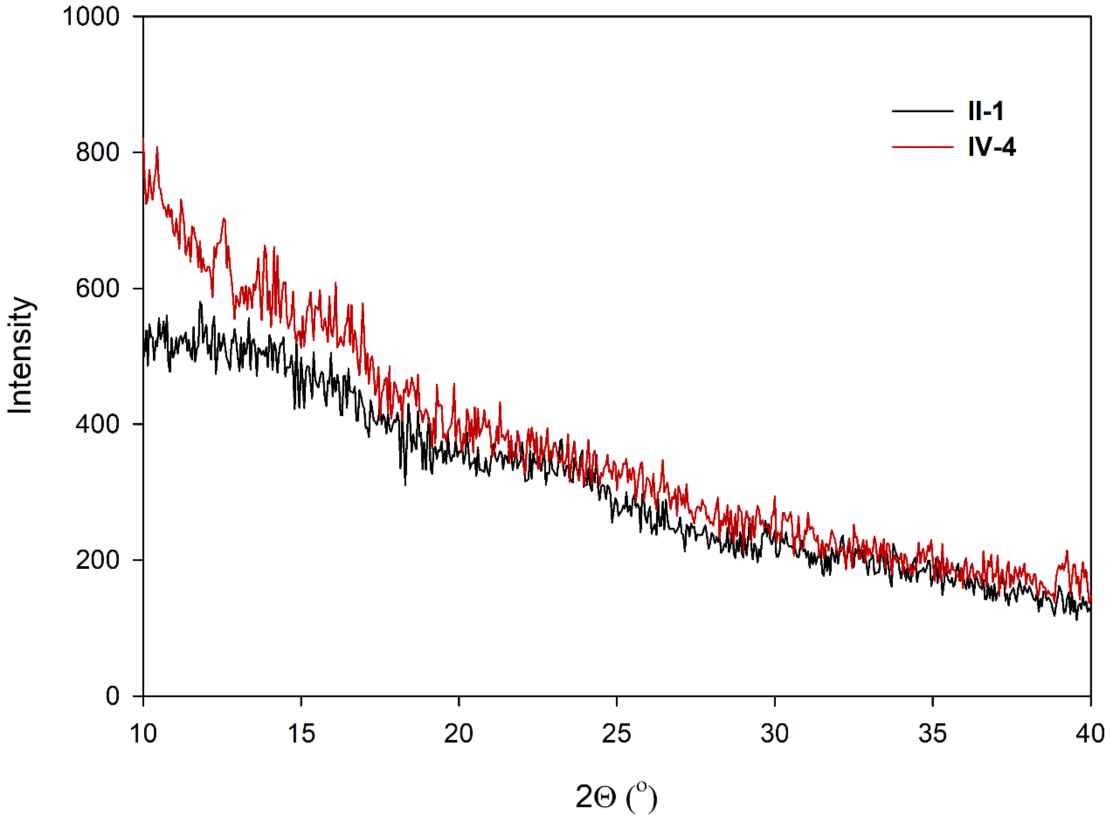

Figure S3. Wide-angle X-ray scattering (WAXS) diffractograms of samples II-1 and IV-4.

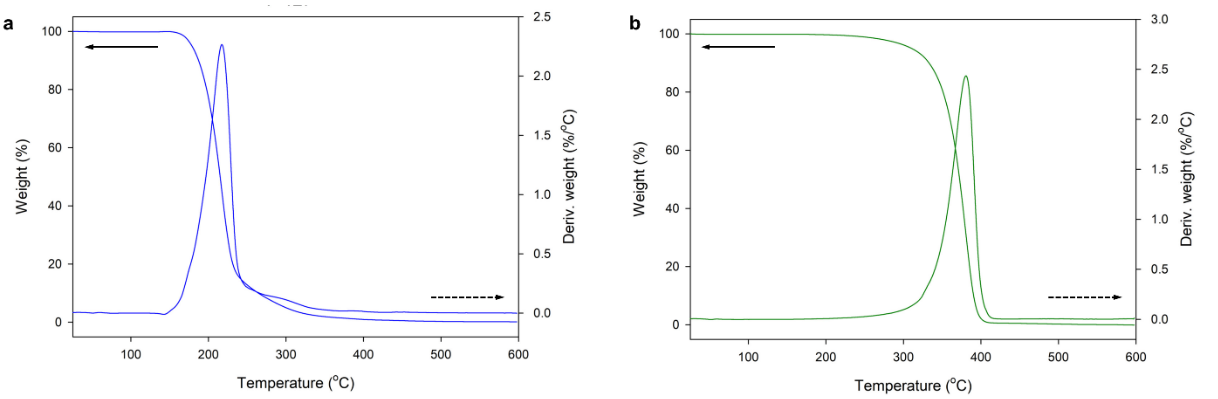

Figure S4. TGA and DTA traces registered for pure Pluronic® F-127 in air (a) and N_2 atmosphere (b) (heating ramp 10 ºC/min) (solid black arrow – TGA, dashed black arrow – DTA).

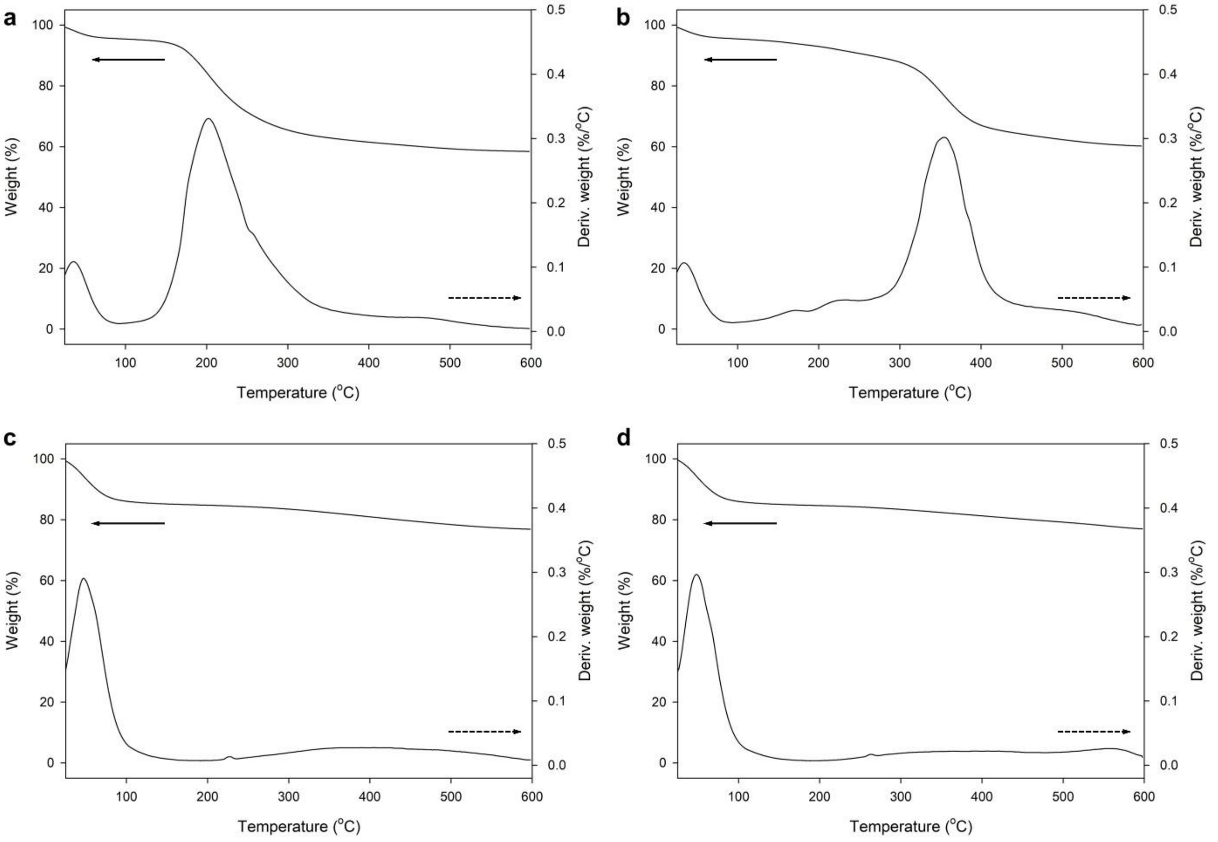

Figure S5. TGA and DTA traces (10 ºC/min) of sample I-5 B-33/14 as prepared (a+b) and after pyrolysis for 2 h at 217 ºC in a furnace (c+d). Analysis in air (a+c) or N_2 (b+d) atmosphere (solid black arrow – TGA, dashed black arrow – DTA).

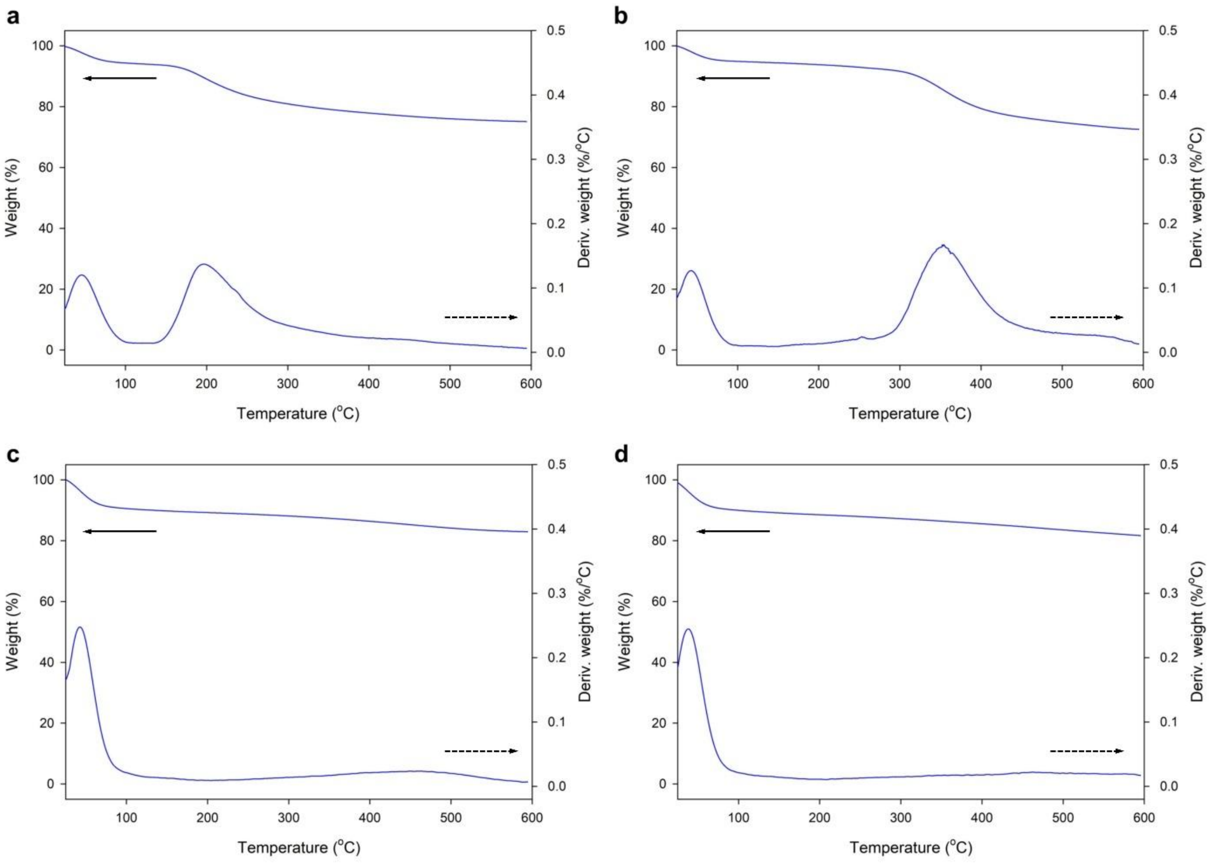

Figure S6. TGA and DTA traces (10 ºC/min) of sample II-1 as prepared (a+b) and after pyrolysis for 2 h at 217 ºC in a furnace (c+d). Analysis in air (a+c) or N_2 (b+d) atmosphere (solid black arrow – TGA, dashed black arrow – DTA).

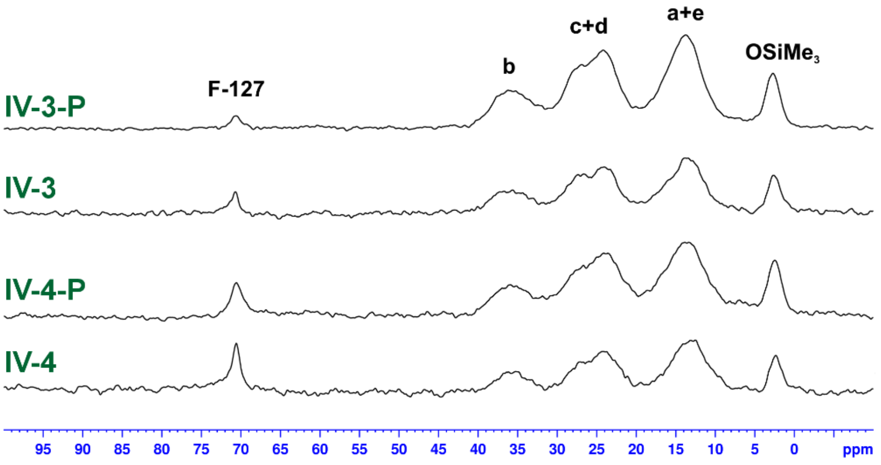

Figure S7. ^{13}C SS NMR spectra (HP Dec) of samples IV-4 and IV-3 before and after the thermal treatment (IV-4-P and IV-3-P, respectively). Symbols a-e indicate different types of methylene protons in LPSQ-R-Si(OMe)$_3$ (as in Figure 2).

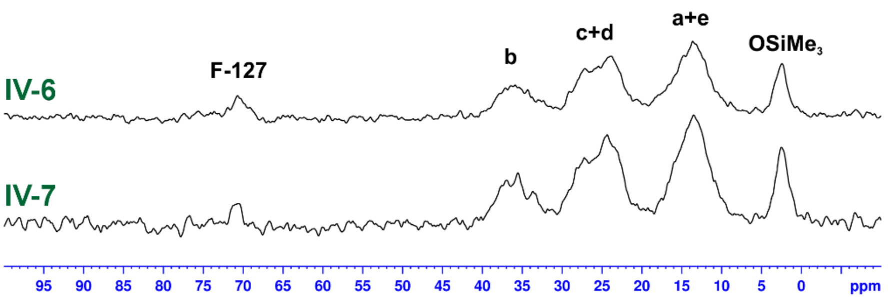

Figure S8. ^{13}C SS NMR spectra (HP Dec) of samples IV-7 and IV-6. Symbols a-e indicate different types of methylene protons in LPSQ-R-Si(OMe)3 (as in Figure 2).

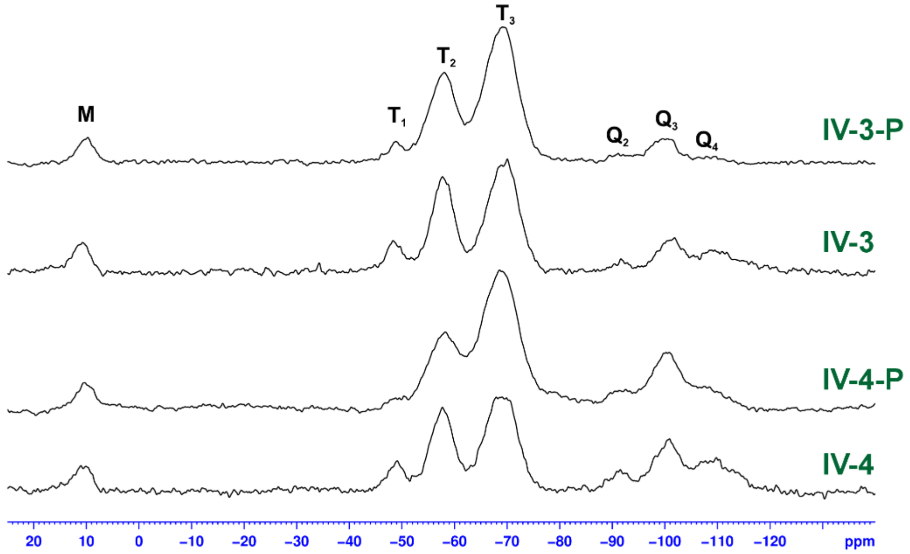

Figure S9. ^{29}Si SS NMR spectra (HP Dec) of samples IV-4 and IV-3 before and after the thermal treatment (IV-4-P and IV-3-P, respectively).

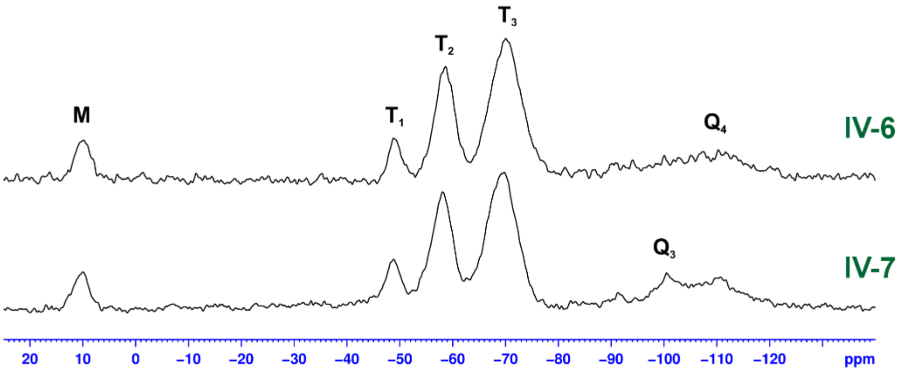

Figure S10. ^{29}Si SS NMR spectra (HP Dec) of samples IV-7 and IV-6.
